# miRNA Biogenesis Enzyme Drosha Is Required for Vascular Smooth Muscle Cell Survival

**DOI:** 10.1371/journal.pone.0060888

**Published:** 2013-04-18

**Authors:** Pei Fan, Zixuan Chen, Peng Tian, Wen Liu, Yan Jiao, Yi Xue, Anindya Bhattacharya, Jianmin Wu, Meifen Lu, Yuqi Guo, Yan Cui, Weikuan Gu, Weiwang Gu, Junming Yue

**Affiliations:** 1 Department of Pathology, University of Tennessee Health Science Center, Memphis, Tennessee, United States of America; 2 Center for Cancer Research, University of Tennessee Health Science Center, Memphis, Tennessee, United States of America; 3 Department of Orthopaedic Surgery–Campbell Clinic, University of Tennessee Health Science Center, Memphis, Tennessee, United States of America; 4 Department of Neurology, University of Tennessee Health Science Center, Memphis, Tennessee, United States of America; 5 Southern Medical University, Guangzhou, Guangdong, P. R. China; 6 The Third Affiliated Hospital of Zhengzhou University, Zhengzhou, Henan, P. R. China; 7 Department of Microbiology, Immunology and Biochemistry, University of Tennessee Health Science Center, Memphis, Tennessee, United States of America; 8 Veterinary Research Institute, Nanning, Guangxi, P. R. China; Centro Cardiologico Monzino, Italy

## Abstract

miRNA biogenesis enzyme Drosha cleaves double-stranded primary miRNA by interacting with double-stranded RNA binding protein DGCR8 and processes primary miRNA into precursor miRNA to participate in the miRNA biogenesis pathway. The role of Drosha in vascular smooth muscle cells (VSMCs) has not been well addressed. We generated Drosha conditional knockout (cKO) mice by crossing VSMC-specific Cre mice, SM22-Cre, with Drosha^ loxp/loxp^ mice. Disruption of Drosha in VSMCs resulted in embryonic lethality at E14.5 with severe liver hemorrhage in mutant embryos. No obvious developmental delay was observed in Drosha cKO embryos. The vascular structure was absent in the yolk sac of Drosha homozygotes at E14.5. Loss of Drosha reduced VSMC proliferation *in vitro* and *in vivo.* The VSMC differentiation marker genes, including αSMA, SM22, and CNN1, and endothelial cell marker CD31 were significantly downregulated in Drosha cKO mice compared to controls. ERK1/2 mitogen-activated protein kinase and the phosphatidylinositol 3-kinase/AKT were attenuated in VSMCs *in vitro* and *in vivo*. Disruption of Drosha in VSMCs of mice leads to the dysregulation of miRNA expression. Using bioinformatics approach, the interactions between dysregulated miRNAs and their target genes were analyzed. Our data demonstrated that Drosha is required for VSMC survival by targeting multiple signaling pathways.

## Introduction

Drosha is an RNAase III enzyme that interacts with the RNA binding protein DGCR8 and forms a microcomplex in the nucleus to process primary miRNAs (pri-miRNAs) into precursor miRNAs (pre-miRNAs), which are subsequently cleaved by Dicer in the cytoplasm to produce mature miRNA. Accumulating experimental evidence suggests that miRNAs control gene expression through RNA-induced silencing complex (RISC) at the posttranscriptional level and regulates cell apoptosis, proliferation, and differentiation [1], [2], [3], [4]. The miRNA biogenesis pathway is tightly controlled through different molecular mechanisms including methylation, RNA editing, and a self-regulatory feedback loop between Drosha and DGCR8 in the physiological condition [5]. Disruption of the miRNA biogenesis pathway causes aberrant expression profiles of miRNA genes, thus leading to various human diseases and developmental retardation.

DGCR8 specifically processes miRNA, whereas Dicer functions in other small RNAs such as siRNA or shRNA. in addition to miRNAs. Recent studies indicated that some splicing-independent mirtron-like miRNA (simtrons) maturation does not require the canonical miRNA biogenesis pathway, which skips the processing of DGCR8, exportin-5, Dicer, and Ago2, but is Drosha-dependent [6], [7], [8]. In our previous studies, we showed that disruption of DGCR8 in VSMCs results in embryonic death 2–3 days earlier than that of Dicer, although both DGCR8 and Dicer cKO embryos share some phenotypic similarity [9], [10]. Our studies demonstrated that DGCR8 plays a more important role than does Dicer in maintaining the VSMC functions by participating in the miRNA process in the upstream biogenesis pathway. Drosha and Dicer are two RNAase III enzymes and share the structural and functional similarity that both cleave the double-stranded RNA and have two RNAase III domains, a/b and an RNA binding domain. However, DGCR8 is an RNA binding protein whose structure differs from Dicer and Drosha, which do not have RNAase III domains but have two RNA binding domains. Drosha, DGCR8, and Dicer are key components of the miRNA biogenesis pathway and contribute to miRNA maturation by participating in this pathway.

In our previous studies [9], [10], we have shown that DGCR8 and Dicer are required for vascular development. However, the roles of Drosha in VSMCs are largely unknown. To investigate the role of Drosha in VSMC function, we generated conditional Drosha knockout mice (cKO) by crossing Drosha ^loxp/loxp^ with VSMC-specific SM22-cre mice. Disruption of Drosha in VSMCs of mice results in embryonic mortality. In addition, we also generated KO VSMCs by using adeno-cre virus and knockdown (KD) VSMCs using retroviral shRNA vector. By characterizing Drosha cKO mice, KO, and KD VSMCs, we found that loss of Drosha in VSMCs reduces VSMC proliferation and differentiation by compromising miRNA biogenesis pathways and attenuating cellular survival pathways ERK1/2 and AKT.

## Results

### Loss of Drosha in VSMCs Results in Embryonic Lethality

**Figure 1:**
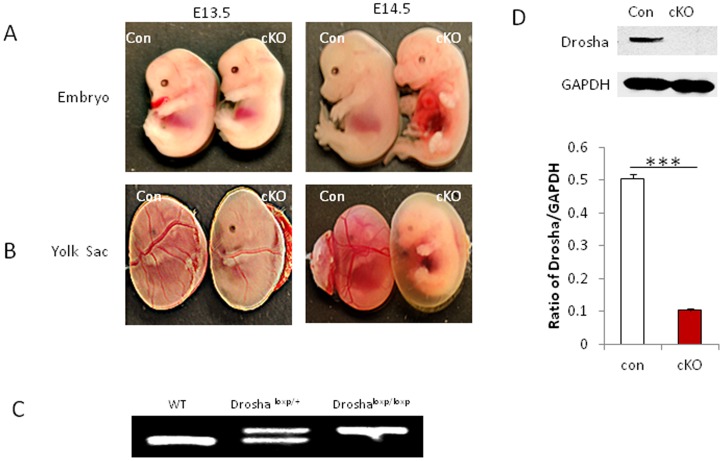
Disruption of Drosha in VSMCs results in embryonic mortality. **A**. Morphology of Drosha cKO (Drosha^loxp/loxp^/SM22-Cre) and control (Drosha^loxp/loxp^) embryos at E13.5 and E14.5. **B**. Vasculature of yolk sac in control and Drosha cKO mice between E13.5 and E14.5. **C**. Genotyping Drosha cKO mice by PCR showed three different genotypes: wild type (WT), heterozygote, and homozygote. **D**. The expression of Drosha in umbilical cord at E13.5 was detected by Western blot in cKO and WT mice (n = 5, ***P<0.001).

To determine the role of Drosha in VSMCs, we generated Drosha cKO mice by crossing Drosha^loxp/loxp^ mice with VSMC-specific SM22-Cre transgenic mice. Exon 9 of Drosha was floxed and deleted through Cre-LoxP recombination, thus leading to disrupting Drosha expression in VSMCs of mice (Figure S1A). Of 120 live pups genotyped, no homozygote Drosha mice were found, suggesting that loss of Drosha in VSMCs of mice results in embryonic lethality. The Drosha heterozygotes were viable and did not display any developmental abnormalities. Drosha homozygotes died at embryonic day E14.5 without obvious developmental delay (Figure 1A). The vasculature was severely impaired in the yolk sacs of Drosha cKO mice after E14.5; no blood vessels were observed (Figure 1B). To further examine the embryonic death of Drosha homozygotes, embryos were collected at different embryonic developmental stages and genotyped by PCR (Figure 1C). Drosha expression was significantly reduced in extracts from the pooled umbilical arteries of control (Drosha^loxp/loxp^) and homozygote (Drosha^loxp/loxp/SM22-cre^) mice as detected by Western blot (Figure 1D).

### Loss of Drosha in VSMCs Leads to Hypoplastic Blood Vessel Walls, Cardiomyopathy, and Liver Hemorrhage

**Figure 2:**
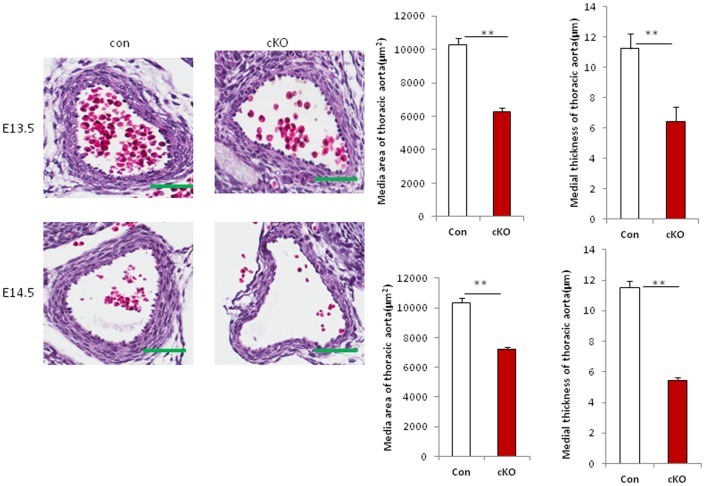
Loss of Drosha in VSMCs results in vascular wall hypoplasia in mice. Sections of thoracic aorta from Drosha cKO and control embryos at E13.5 and E14.5 were stained with hematoxylin and eosin. The medial area and thickness of blood vessel walls of Drosha cKO mice and littermate controls were measured using the Elements image analysis program (Nikon) in three sections from each embryo. The media area of the vessel and the wall thickness were calculated from the inner and outer media circumference of vessel walls. Error bars indicate standard deviation (SD). Four different embryos were analyzed. Scale bars indicates 50 µm (**P<0.01; ***P<0.001).

**Figure 3:**
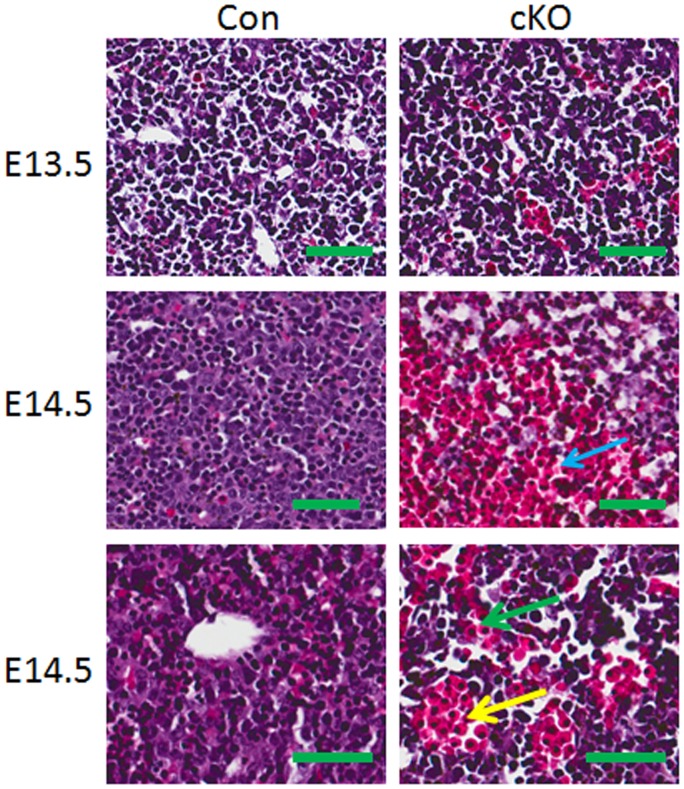
Drosha VSMC-specific cKO mutants display severe liver hemorrhage. Sections from Drosha cKO and littermate control embryos at E13.5 and E14.5 were stained with H&E. The red blood cells occupied the hepatic plate (blue arrow), hepatic vein (yellow arrow), and sinusoids (green arrow) in the liver (scale bars represent 50 µm).

Embryos were collected at E13.5 and E14.5, and histological analysis was performed. The media area and thickness of the thoracic aorta of Drosha cKOs were significantly reduced in Drosha cKO mice compared to controls, indicating that loss of Drosha in VSMCs leads to vascular wall hypoplasia (Figure 2). Previous studies have indicated that SM22 promoter is also active in cardiomyocytes during embryonic developmental stages [9], [10]. We found that the heart was enlarged in Drosha cKO embryos relative to controls with reduced ventricular walls, which is a feature of cardiomyopathy (Figure S1B). Liver hemorrhage was observed in Drosha cKO embryos at E14.5. In some regions of the liver, the hepatic structure was completely disrupted and red blood cells were accumulated without hepatocytes. In addition, red blood cells were congested in the hepatic artery, the portal vein, and sinusoids (Figure 3).

### Disruption of Drosha in VSMCs Reduced Cell Proliferation without Inducing Apoptosis

**Figure 4:**
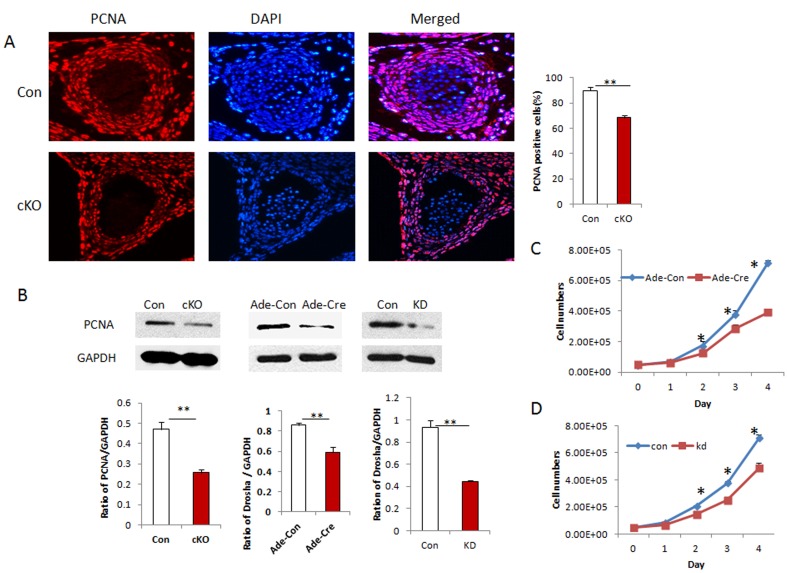
Disruption of Drosha in VSMCs of mice reduces cell proliferation. **A.** Paraffin-embedded sections of thoracic aorta at E13.5 from control and cKO embryos were immunostained with the proliferating cell marker PCNA, and cell nuclei were counterstained with DAPI. The proliferating cells were counted and divided by the total number of nuclei as the proliferating index. Four different embryos were analyzed (error bar represent standard deviation; **P<0.01). **B.** VSMC proliferations in the umbilical arteries. KO VSMCs and KD VSMCs were examined using Western blot. The band intensity was normalized to GAPDH, and the ratios were used to analyze significant difference (**p<0.01, ***P<0.001). **C.** VSMC proliferation rates at different time points were examined by cell counts in Drosha KO and control VSMCs; rates were established by transducing Ade-Cre and Ade-Con viruses, respectively. Three separate experiments were performed, and significant differences were analyzed at each time point between control and KO cells (**P<0.01). **D.** Cell proliferations in KD and control VSMCs generated using retroviral knockdown vector were examined by counting cell numbers. Triplicate experiments were performed, and significant differences were analyzed between control and KD VSMCs (**P<0.01).

To determine whether hypoplasia of blood vessel walls in Drosha cKO arteries was caused by reduced VSMC proliferation or increased apoptosis, we stained sections of thoracic aorta of E13.5 embryos by using anti-PCNA antibody. VSMC proliferation was significantly reduced in Drosha cKO embryos compared with controls (Figure 4A). PCNA expression in umbilical arteries of E13.5 Drosha cKO embryos was also detected by Western blots, which showed a significant reduction. In addition, we generated VSMC KO cells by transducing Ade-Cre and Ade-control adenoviruses into VSMCs isolated Drosha^loxp/loxp/SM22-cre^ mice. We also generated Drosha knockdown (KD) VSMCs transduced with retroviral shRNA vector. VSMC proliferation was examined by Western blot in KO and KD VSMCs by detecting PCNA expression; knockout or knockdown of Drosha significantly reduces VSMC proliferation was further verified (Figure 4B). Meanwhile, we also examined cell proliferation by counting cell numbers in KO and KD VSMCs. Similarly, we found that KO or KD of Drosha in VSMCs inhibits cell growth *in vitro* (Figure 4C, D). To examine whether loss of Drosha induces apoptosis in VSMCs of Drosha cKO embryos, sections from the thoracic aorta of E13.5 embryos were examined by TUNEL staining. No apoptosis in the media area of thoracic aortas was observed (data not shown). Taken together, those data indicate that disruption of Drosha expression in VSMCs reduces cell proliferation *in vitro* and *in vivo* without inducing apoptosis.

### Loss of Drosha in VSMCs Reduces VSMC Marker Gene Expression

**Figure 5:**
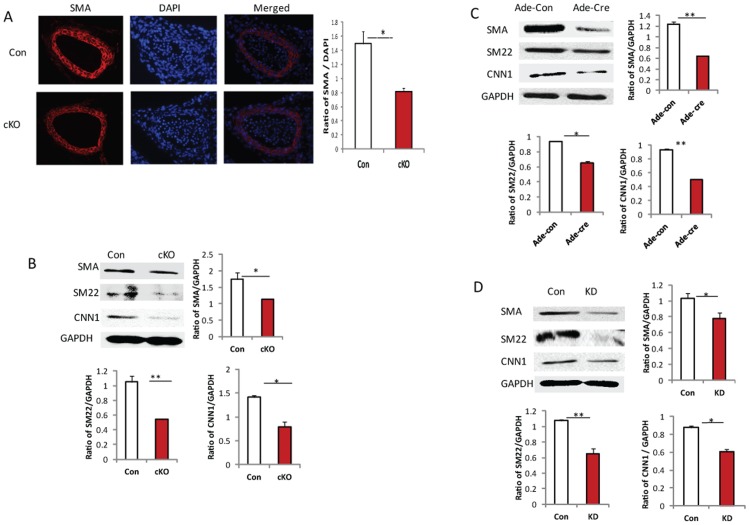
VSMC differentiation marker genes were downregulated in Drosha cKO mice. **A**. Sections of thoracic aorta of control and Drosha cKO at E13.5 were stained with αSMA antibody. **B**. The expressions of αSMA, SM22, CNN1, and GAPDH in umbilical arteries of control and cKO mice at E13.5 were detected by Western blot. Data represent mean ± SD (n = 5). **C.** VSMC marker genes in KO and control VSMCs generated using Ade-con and Ade-Cre viruses were detected by Western blot. Three separate experiments were performed. **D.** VSMC marker genes in KD and control VSMCs were examined using Western blot. Protein bands were quantified by densitometry for analysis of significant differences (*P<0.05,**p<0.01, ***P<0.001).

The contractile phenotype of VSMC is regulated by the expression of differentiation marker genes, including αSMA, SM22, and CNN1. To determine whether Drosha regulates VSMC differentiation, using αSMA antibody we stained sections of the thoracic aorta of Drosha cKO embryos at E13.5 and Drosha KO as well as KD VSMCs. Our results showed that loss of Drosha leads to significant downregulation of αSMA in VSMCs of Drosha embryos (Figure 5A), KO (Figure S2), and KD VSMCs (Figure S3A) compared with controls. Drosha knockdown was also observed in VSMCs transduced with retroviral shRNA vector following immunostaining KD cells (Figure S3B). The expression of VSMC differentiation marker genes, αSMA, SM22, and CNN1, in umbilical arteries of E13.5 embryos was detected using Western blots, which showed significant downregulation (Figure 5B). Similarly, these marker genes were also significantly downregulated in Drosha KO and KD VSMCs (Figure 5C, D). To examine whether VSMC specific miR-145 contributes to the downregulation of VSMC marker genes, we further transduced Drosha KD cells using miR-145 lentiviral vector and found that the downregulation of VSMC marker genes can be partially rescued by re-introducing miR-145 expression(Figure S4). To examine whether disruption of Drosha in VSMCs affects endothelial cell (EC) function, we stained sections of thoracic aorta from E14.5 Drosha cKO and control embryos by using EC specific antibody CD31, whose expression was significantly reduced in Drosha cKO embryos compared to that in controls (Figure S5A). The expression of CD31 in the umbilical arteries of Drosha cKO embryos was significantly reduced compared to that in controls, as detected by Western blot (Figure S5B).

### Disruption of Drosha in VSMCs Attenuates ERK1/2 and PI3K/AKT Pathways

**Figure 6:**
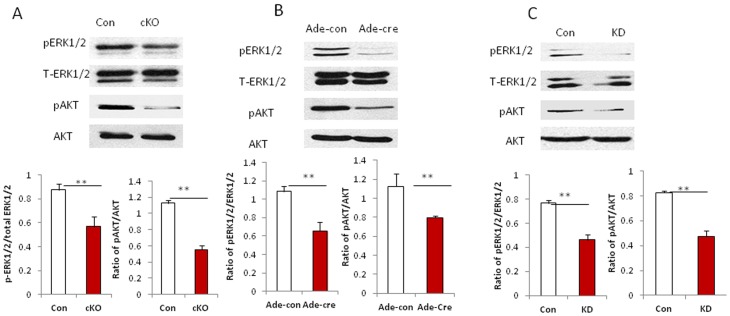
Disruption of Drosha in VSMCs attenuates ERK1/2 and AKT *in vitro* and *in vivo*. **A.** Phospho and total ERK1/2 and AKT were detected by Western blot in umbilical arteries pooled from embryos of Drosha cKOs and WT controls at E12.5 (n = 6). **B.** The expression of phospho and total ERK1/2 and AKT in knockout and control VSMCs were detected by western blot. Protein band intensity was quantified using densitometry and normalized to GAPDH. (*p<0.05,**P<0.01,***P<0.001). **C.** The expression of phospho and total ERK1/2 and AKT in KD and control VSMCs was detected by Western blot. Error bars indicate the SD (**P<0.01).

To determine whether disruption of the Drosha-mediated miRNA biogenesis pathway affects cellular survival pathways in Drosha cKO mice, we examined the levels of activated ERK1/2 and AKT by Western blot in umbilical arteries of Drosha KO in E13.5 embryos. We found that both pathways were attenuated in umbilical arteries of Drosha cKO embryos compared with control embryos (Figure 6A). We also examined the two pathways in KO and KD VSMCs. Similarly, both ERK1/2 and AKT was attenuated in Drosha KO and KD VSMCs compared to controls (Figure 6B, C). Our data demonstrate that loss of Drosha in VSMCs attenuates the cellular survival pathways ERK1/2 and AKT by disrupting the miRNA biogenesis pathway.

### miRNA Expression Profile is Dysregulated in Drosha cKO Mice

**Figure 7:**
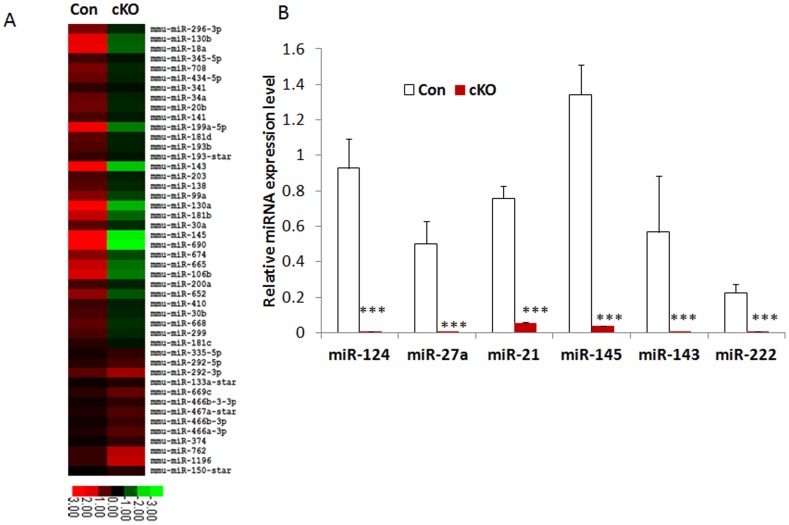
miRNA expressions were dysregulated in Drosha cKO mice. **A.** miRNA expression profiles of Drosha cKO mutant compared to controls detected by miRNA array; the heatmap was generated with those miRNAs whose expressions were down- or upregulated more than 2-fold compared to controls. **B**. Relative miRNA expression levels were detected by performing real-time RT-PCR (***P<0.001).

**Figure 8:**
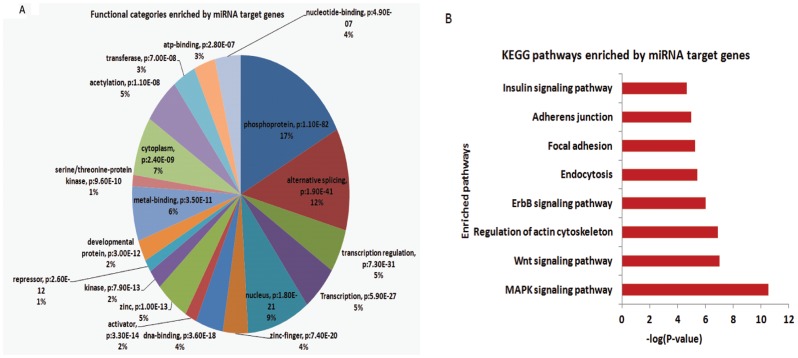
Bioinformatics analysis of dysregulated gene and pathways in Drosha cKO mice. **A.** Functional categories enriched by miRNA target genes. Percentages of target genes in the functional categories were proportionally displayed. **B.** KEGG pathways enriched by miRNA target genes.

Drosha cleaves double-stranded primary miRNA in the nucleus by interacting with the double-stranded RNA binding protein DGCR8 through the miRNA biogenesis pathway. Disruption of Drosha would be expected to dysregulate miRNA expression. To determine the role of Drosha on the expression of miRNA in VSMCs, we performed a miRNA array by extracting RNA from umbilical arteries pooled from E13.5 embryos of controls and Drosha cKO mice. Of 670 mouse miRNA probes in the array, after filtering miRNAs with low signals, 198 miRNAs were detected; 104 showed significant downregulation, 27 were not significantly altered, and 67 were upregulated in Drosha cKOs compared with controls (Table S2). 46 miRNAs with at least 2-fold up- or downregulation were clustered and displayed in a heat map (Figure 7A). Based on the miRNA array data, several miRNAs linked with VSMC functions in previous studies were selected to be further verified by performing real-time RT-PCR. VSMC-specific miR-143/145 cluster modulates a VSMC phenotypic switch between proliferation and differentiation [11], [12], [13], [14]. We found that, in the umbilical arteries of Drosha cKO embryos, the expression of miR-143 and -145 decreased ∼124- and 35-fold compared to controls, respectively (Figure 7B). miR-21 was implicated in regulating VSMC proliferation in a previous study, which was approximately a 14-fold reduction, whereas miR-222, a VSMC proliferative miRNA, was reduced ∼27-fold in Drosha cKO embryos compared to controls. Our data indicate that miRNA expressions were dysregulated in Drosha cKO mice following disrupting Drosha in VSMCs of mice. 46 miRNAs with at least a 2-fold up- or downregulation compared to controls were selected, and their 3,890 target genes were analyzed using DAVID. We found that 19 functional categories were significantly enriched (Figure 8A). The two most significantly enriched functional categories were the phosphoprotein, *P* − = 1.1×10^−82^, and the alternative splicing, *P* = 1.9×10^−41^ (Figure 8A). We further analyzed the enrichment of KEGG pathways for the target genes. Multiple pathways were affected by the target genes including MAPK, WNT, and actin-cytoskeleton regulation pathways (Figure 8B).

## Discussion

### Drosha cKO Shares Phenotypic Similarity to DGCR8 and Dicer VSMC-specific cKO Mice

Loss of Drosha in VSMCs resulted in multiple embryonic defects including severe liver hemorrhage, vascular wall hypoplasia, and embryonic lethality. The Drosha cKO mutants died at E14.5, which is 2 days earlier than Dicer cKO mice died, but 2 days later than DGCR8 cKO mice died (Table S1) [9], [10]. However, embryonic developmental delay was observed in both Dicer and DGCR8 cKO mice but not in Drosha cKO mice. Interestingly, a similar study showed that deletion of exon 21 to 22 of Dicer in VSMCs leads to liver hemorrhage and embryonic death at E17.5; growth delay was not observed [15]. Compared to controls, Drosha cKO embryos showed dilated ventricular wall structure, which was partly contributed by disrupting Drosha in cardiomyocytes as a result of the activity of SM22 promoter during embryonic stage. Loss of Drosha in VSMCs leads to the downregulation of endothelial cell marker gene CD31 expression, indicating that EC functions were also partially disrupted through VSMC and EC interaction. Therefore, both cardiomyopthy and disrupted vasculature contributed to embryonic lethality of Drosha cKO mice. Drosha and Dicer are RNAase III enzymes that cleave double-stranded RNA, whereas DGCR8 is an RNA binding protein that interacts with Drosha to stabilize its cleavage in the miRNA biogenesis pathway. The phenotypic similarities observed from all three cKO mutants indicate that Drosha, DGCR8, and Dicer share a functional similarity, implicating their similar roles in the miRNA biogenesis pathway by contributing to miRNA maturation. Therefore, our data suggest that phenotypic similarities among Drosha, DGCR8, and Dicer may be partially caused by disrupting the miRNA biogenesis pathway.

The differences observed from three VSMC-specific cKO mice were the timing and severity of embryonic death: DGCR8 was the earliest and severest, followed by Drosha, and then Dicer. Drosha and DGCR8 function in cell nuclei to process pri-miRNA into pre-miRNA upstream of the miRNA biogenesis pathway, whereas Dicer cleaves pre-miRNA into mature miRNA in the cytoplasm downstream of the miRNA biogenesis pathway. Disruption of Drosha or DGCR8 might impair miRNA maturation at an earlier step than that of Dicer. Therefore, Drosha- and DGCR8-mediated miRNA biogenesis pathway may play a more important role than that of Dicer in VSMCs. However, Dicer functions not only on miRNA process but also on other small RNAs including siRNA and shRNA. A previous study showed that Dicer cKO mice showed a more severe phenotype than that of DGCR8 when both genes were individually deleted in postmitotic neurons. This difference attributed to that loss of Dicer in neurons resulted in the Dicer-dependent loss of mirtrons and H/ACA snoRNA-derived small RNAs, which are DGCR8 independent [7]. Therefore, to understand the phenotypic difference of Drosha compared to DGCR8 and Dicer, further studies are required to investigate the functions of individual miRNAs enriched from Drosha cKO mice by using loss or gain of function approaches *in vitro* and *in vivo.*

### Drosha Shares Functional Similarity to DGCR8 and Dicer in VSMCs

In this study, we showed that loss of Drosha reduces VSMC proliferation. Similarly, we found that loss of DGCR8 or Dicer in VSMCs also inhibits cell proliferation as reported previously [9], [10]. However, our studies demonstrated that loss of Drosha or Dicer does not induce VSMC apoptosis in the wall of blood vessels, whereas loss of DGCR8 does. These findings suggest that DGCR8 plays a more important role in VSMCs than that of Drosha or Dicer, both of which demonstrated a distinct role in maintaining cellular homeostasis by regulating cell proliferation and apoptosis.

Loss of Drosha in VSMCs inhibits VSMC marker gene expression, a similar finding in DGCR8 and Dicer VSMC-specific cKO mice [9], [10]. Therefore, disruption of the miRNA biogenesis pathways by deleting Drosha, DGCR8, or Dicer reduces VSMC marker gene expression, subsequently regulating cell contraction, which has been shown in our DGCR8 knockout VSMCs [9] and Dicer-inducible VSMC-specific knockout mice [16]. So far, the molecular mechanism of how Drosha, DGCR8, and Dicer regulate cell proliferation and differentiation is still unknown. However, miR-143 and miR-145 were significantly downregulated in Drosha KO VSMCs compared to controls. Previous studies showed that miR-143/145 contributed to the VSMC phenotypic modulation by enhancing VSMC marker gene expression including SMA, SM22, and CNN1 [12], [17]. Our data indicate that downregulation of VSMC marker genes in Drosha KD cells can be partially rescued by introducing miR-145 expression, suggesting that loss of miR-145 in Drosha KO partially contributes to VSMC marker gene reductions.

### Loss of Drosha in VSMCs Attenuates the Cell Survival Pathways ERK1/2 and AKT

Previous studies showed that the miRNA pathway crosstalks with the TGFβ cellular pathway [18]. We also showed in recent studies that disruption of Dicer or DGCR8 in VSMCs attenuated two cell survival pathways ERK1/2 and AKT [9], [10]. Similarly, we found that loss of Drosha attenuates both pathways in VSMCs *in vitro* and *in vivo.* Our data from Drosha, DGCR8, and Dicer cKO mice suggest that disruption of the miRNA biogenesis pathway by deleting Drosha, DGCR8, or Dicer impairs the miRNA biogenesis pathway, subsequently attenuates cellular survival pathways, thus reducing cell proliferation. In our previous work, we showed that loss of DGCR8 leads to significant downregulation of the miR-17/92 cluster in VSMCs of DGCR8 cKO mice compared to controls, which may partially contribute to reduced cell proliferation by attenuating ERK1/2 and AKT pathways. Previously, we showed that miR-21 activates the PI3/AKT pathway by targeting PTEN in VSMCs [19]. miR-21 was also shown to activate the ERK1/2 pathway by targeting Sprout 1/2 in VSMCs [20], [21]. However, miR-21 is not the most downregulated miRNA in Drosha cKO mice, although it was significantly downregulated as we confirmed by real-time RT-PCR. It is not clear if dysregulated miRNAs directly regulate ERK1/2 and PI3K/AKT pathways; further investigation is required. Our bioinformatics analysis indicates that loss of Drosha in VSMCs leads to the downregulation of the majority of miRNAs and dysregulation of multiple signaling pathways including the MAPK and WNT pathways.

### miRNA Expression Profiles were Dysregulated in Drosha cKO Mice

Our current study indicates that disruption of Drosha in VSMCs of mice dysregulated miRNA expression, suggesting that Drosha plays a key role in the miRNA biogenesis pathway. Although miRNA expression profiles were altered in Drosha cKO mice compared to controls, those dysregulated miRNAs are slightly different from miRNAs in DGCR8 or Dicer VSMC-specific cKO mice, as we published previously [9], [10]. However, some dysregulated miRNAs in Drosha cKO mice are the same as in DGCR8 and Dicer cKO mice (e.g., the miR-143/145 cluster was enriched as the VSMC-specific miRNA and downregulated in all three cKO mice). The individual miRNAs of miR-17/92 cluster were all downregulated in DGCR8, whereas only miR-17, -18a, and -20b from this cluster were significantly downregulated in Drosha cKO mutants. In Dicer cKO mutants, miR-18, -19, and- 92a were downregulated, whereas miR-17 and -20a were upregulated. Although we performed miRNA array analysis and identified those dysregulated miRNAs whose functions have not been well investigated yet. For example, the functions of miR-130b, -708, -199a in VSMCs are completely unknown, although they were highly expressed in VSMCs and downregulated in Drosha cKO mutant embryos based on miRNA array data. The upregulation of miRNAs in gastrointestinal smooth muscle specific Dicer KO mice was also reported previously [22]. Moreover, recent studies also showed that Dicer is not required for some miRNA maturation. For example, miR-451 maturation is Dicer independent but Ago2 dependent [23], [24]. Some miRNA (simtron) maturations do not require DGCR8 or Dicer but do require Drosha (e.g., miR-1225 and miR-1228) [6]. Some miRNAs (mitrons) such as miR-877, miR-1224, and miR-1226 are independent of the canonical miRNA biogenesis pathway but dependent on the splicing process [25]. Taken together, those studies indicate that Drosha, DGCR8, and Dicer are required for the majority of miRNA maturation by participating in the miRNA biogenesis pathway. Some individual miRNA maturations are not fully dependent on the canonical miRNA biogenesis pathway but require one of them or none at all in a cell- or tissue-specific manner. Therefore, future studies are required to understand the maturation pathway of individual miRNA or whether other small RNAs including siRNA or shRNA play a role in the functions of Drosha, DGCR8, or Dicer in a specific cell or tissue manner. The phenotype of Drosha cKO was caused by the dysregulation of miRNA and gene network, not the individual miRNA. In our previous study, we showed that disruption of DGCR8 in VSMCs leads to downregulation of the miR-17/92 cluster. Although loss of DGCR8 in VSMCs leads to embryonic lethality, our recent work indicates that deletion of the miR-17/92 cluster in VSMCs does not lead to any developmental abnormality in mice(Figure S6). Moreover, another study indicates that loss of DGCR8 dysregulated multiple mRNAs and other small nucleolar RNAs (snoRNA) [26]. Our bioinformatics analysis also showed that dysregulated miRNA disrupted multiple signaling pathways. To elucidate the phenotype of Drosha cKO, it is required to construct the miRNA-gene network by performing gene array analysis, then globally analyzing the miRNA/mRNA interaction. Therefore, we conclude that loss of Drosha in VSMCs leads to embryonic lethality by globally disrupting the miRNA/mRNA regulatory network.

## Materials and Methods

### Primary VSMC Isolation and Cell Culture

Primary VSMCs were isolated from 2-month-old Drosha^loxp/loxp^ mice and immortalized using SV40 large T antigen, then transduced with Ade-cre and Ade-con adenoviruses to generate Drosha knockout and control VSMCs, respectively. Primary VSMCs were characterized by immunostaining with αSMA antibody. Mouse VSMCs were obtained from ATCC and cultured in Dulbecco’s Modified Eagle Medium (DMEM) supplemented with 10% FBS (Hyclone; Logan, UT), 100 U/ml penicillin, and 100 µg/ml streptomycin (Invitrogen, Carlsbad, CA).

### Generation of VSMC Drosha cKO Mice

All animal procedures were performed in accordance with the Guide for the Care and Use of Laboratory Animals and approved by the Institutional Animal Care and Use Committee at the University of Tennessee Health Science Center (Protocol number: 1971). Mice were anesthetized by intraperitoneal injection of Avertin (0.75 mg/g body weight) and euthanized by cervical dislocation prior to tissue collection. VSMC Drosha cKO mice were generated by crossing SM22-Cre mice (Jackson Laboratory, Stock #004746) with Drosha^loxp/loxp^ mice (Jackson Laboratory, Stock #008592). Drosha^loxp/loxp^/SM22-Cre mice were generated by intercrossing Drosha^loxp/+^/SM22-Cre mice or breeding Drosha^loxp/loxp^ mice with Drosha^loxp/+^/SM22-Cre mice. Drosha^loxp/loxp^ littermates were used as controls for all experiments. All mice used in this study were on C57BL/6 (B6) genetic background. miR-17/92 VSMC specific cKO mice were generated by crossing miR-17/92^loxp/loxp^(Jackson Laboratory, Stock #008459) with SM22-Cre mice. miR-17/92 cKO and control mice are maintained in B6/129S4 mixed genetic background.

### Genotyping Drosha cKO Mice and Embryos

Mouse genomic DNA extraction was performed as previously described [9].

### Histological Analysis

To analyze the phenotype of Drosha cKO embryos, time-mated pregnant females were sacrificed, and embryos were collected. The harvested embryos were weighed and fixed overnight in 4% paraformaldehyde, embedded in paraffin, sectioned, and stained with H&E.

### Retroviral and Lentiviral Vector Production

Retroviral shRNA vectors against Drosha were purchased from Addgene. Scramble shRNA contains a hairpin sequence targeting luciferase. The retroviruses were produced in GD-293 package cell line (Clonetech) by co-transfecting pCMV-VSVG and shRNA vectors by using superfect (Qiagen). The supernatant was collected 60 h following transfection and purified by ultracentrifugation for 90 mins at 25000 g. miR-145 and control lentiviral vectors were produced as described previously [9].

### Immunofluorescence Staining

To detect the VSMC proliferation and differentiation, sections or cells were used to detect the proliferating cell nuclear antigen (PCNA) or α-smooth muscle actin (αSMA) as described previously [9].

### Western Blotting

Umbilical cords or VSMCs were collected in RIPA buffer (Thermo Scientific; Rockford, IL) containing 1% Halt proteinase inhibitor Cocktail (Thermo Scientific). Drosha, SM22 (Santa Cruz; Santa Cruz, CA), β-actin, CNN1, αSMA, and GAPDH (Sigma; St. Louis, MO), pERK, pAKT, ERK1/2, AKT (Cell Signaling; Danvers, MA), or PCNA (Vector Laboratories, Inc.; Burlingame, CA) were detected as described previously [9].

### Affymetrix miRNA Array and Data Analysis

Total RNA was isolated from umbilical arteries by using Trizol Reagent (Invitrogen; Carlsbad, CA), and RNA was further purified by RNeasy MinElute Cleanup Kit (Qiagen; Valencia, CA). The quality and integrity of the total RNA was determined with an Agilent bioanalyzer. The miRNA microarray profiling was performed using Affymetrix GeneChip miRNA arrays (Santa Clara, CA). One µg of total RNA was labeled by polyA polymerase addition with the Genisphere FlashTag HSR kit following the manufacturer's instructions (Genisphere; Hatfield, PA). Labeled RNA was hybridized to the Affymetrix miRNA array 1.0. Chips were washed, stained in a Fluidic Station 450, and scanned (Scanner 3000 7G; Affymetrix). Expression profiles of miRNA between umbilical arteries of Drosha cKO and controls were compared, and all the miRNAs with at least 2-fold changes were selected. Mature miRNA sequences were obtained from miRBase release 19 [27]. Base 2 to 7 of the mature miRNA sequences were collected as seed regions. 3′UTR sequences of the mouse genome were downloaded from the UCSC table browser [28]. PERL codes for TargetScan 6.2 were used to predict the target sites of the selected miRNAs from the complementary sequence matches between miRNA seeds and 3′UTRs of the genes [29]. Sets of genes with at least one miRNA target site in their 3′UTRs were selected for functional association study. DAVID v6.7 was used to predict the enriched functional categories and enriched signaling pathways for the set of target genes [30]. DAVID computes the Fisher exact P values for gene ontology (GO) categories (i.e., Biological Process, Cellular Component, and Molecular Function), and signaling pathways in the KEGG [31] database. We considered GO categories with *P* values less than 5.00E−07 as enriched. For signaling pathways, *P* values less than 1.00E−04 were reported as significant.

### Statistical Analysis

Data shown are the mean ± standard deviation (SD) from at least three different experiments. The differences were analyzed using Student’s *t-*test. P values <0.05 were considered significant.

## Supporting Information

Figure S1 **Conditional Inactivation of Drosha in Mouse VSMCs by Gene Targeting. A.** The Drosha^loxp/loxp^ allele contains loxP sites flanking exon 9. In the SM22-Cre VSMC-specific Cre transgenic mouse line, Cre recombinase expression is driven by the SM22 promoter. **B.** High-power view of left and right ventricular free wall at E14.5. Scale bar = 50 µm.
(PDF)

Figure S2 **Immunostaining of SMA in KO VSMCs.** The expressions of SMA in Drosha KO and control VSMCs generated using Ade-con and Ade-Cre were examined by immunostaining using SMA antibody; the significant differences were analyzed from four separate experiments by quantifying the fluorescent intensity in the Drosha KO VSMCs compared with controls (*P<0.05).
(PDF)

Figure S3 **Immunostaining of SMA and Drosha in KD VSMCs.** **A.** The expressions of SMA in Drosha KD and control VSMCs generated using retroviral shRNA vector were examined by immunostaining using SMA antibody; the significant differences were analyzed from three separate experiments by quantifying the fluorescent intensity in the Drosha KO VSMCs compared with controls (**P<0.01). **B.** The expressions of Drosha in KD and control VSMCs were examined using immunostaining and Western blot. Three separate experiments were performed, and significances were analyzed (**P<0.01).
(PDF)

Figure S4 **Rescue VSMC marker gene expressions in Drosha KD VSMCs.** Drosha KD and control VSMCs were transduced using miR-145 and control lentiviral vector, respectively. The expressions of VSMC marker genes including SMA, SM22, and CNN1 were detected using Western blot. The significant differences were determined by measuring band intensity and calculated from three separate experiments (*P<0.05).
(PDF)

Figure S5 **Immunostaining and immunobloting of CD31 in thoracic aorta. A.** The thoracic aorta of Drosha cKO and control embryos at E14.5 were stained with endothelial cell marker CD31 (*P<0.05). **B**. The expression of endothelial cell marker CD31 was examined in umbilical arteries of E14.5 in Drosha cKO and control embryos by Western blot (**P<0.01)
(PDF)

Figure S6 **miR-17/92 VSMC-specific KO mice are developmentally normal. A.** The bodyweight of 1-month-old miR-17/92 VSMC cKO mice were compared with that of controls (n = 6, NS: no significance ). **B.** Three different genotypes of miR-17/92 cKO mice were detected by PCR.
(PDF)

Table S1 **Phenotypic comparison of VSMC-specific Drosha, DGCR8, and Dicer cKO mice.** Phenotypic differences among Drosha, DGCR8, and Dicer cKO were compared to controls and summarized in Table 1.
(PDF)

Table S2 **miRNA expression profiles in the umbilical arteries of Drosha cKO and control embryos.** miRNA array was performed using RNA samples extracted from umbilical arteries of E13.5 Drosha cKO and control embryos (n = 6). miRNA expressions and *P* values for both Drosha cKO and controls.
(PDF)
