# Supplementary figures and images for: miRNA Biogenesis Enzyme Drosha Is Required for Vascular Smooth Muscle Cell Survival
Source: PLoS One. 2013 Apr 18;8(4):e60888. doi: 10.1371/journal.pone.0060888 (PMC3630177; doi:10.1371/journal.pone.0060888)

A

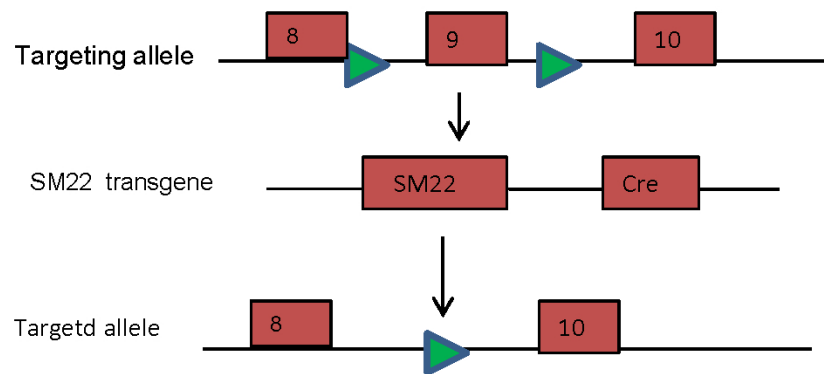

B

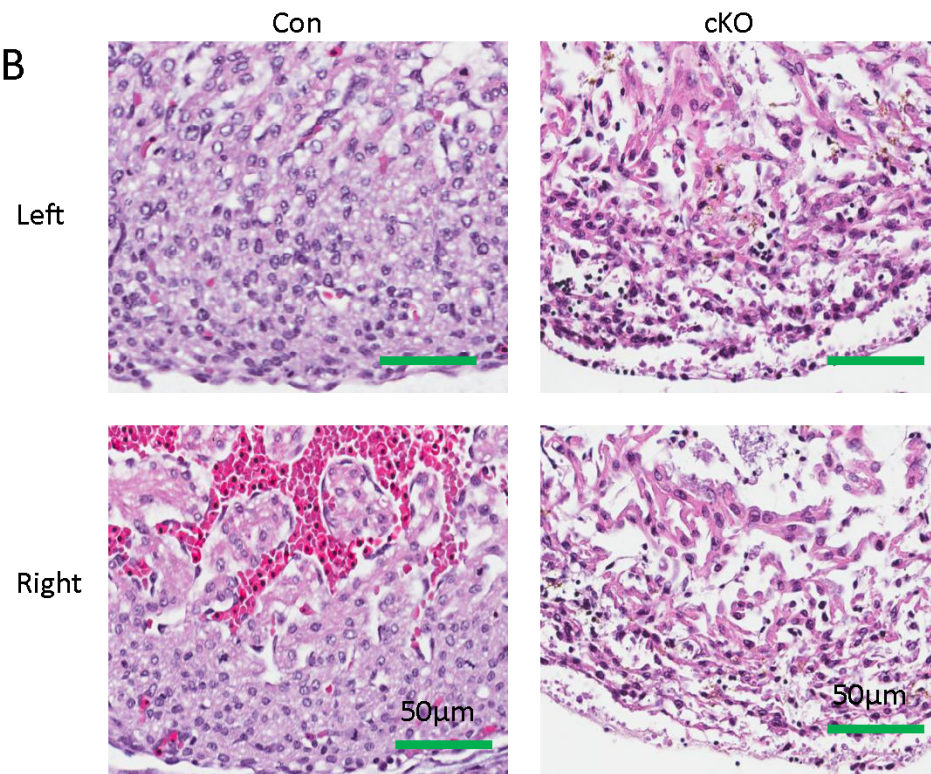

Supplement: Figure S1 — Conditional Inactivation of Drosha in Mouse VSMCs by Gene Targeting. A. The Droshaloxp/loxp allele contains loxP sites flanking exon 9. In the SM22-Cre VSMC-specific Cre transgenic mouse line, Cre recombinase expression is driven by the SM22 promoter. B. High-power view of left and right ventricular free wall at E14.5. Scale bar = 50 µm. (PDF) [file pone.0060888.s001.pdf]

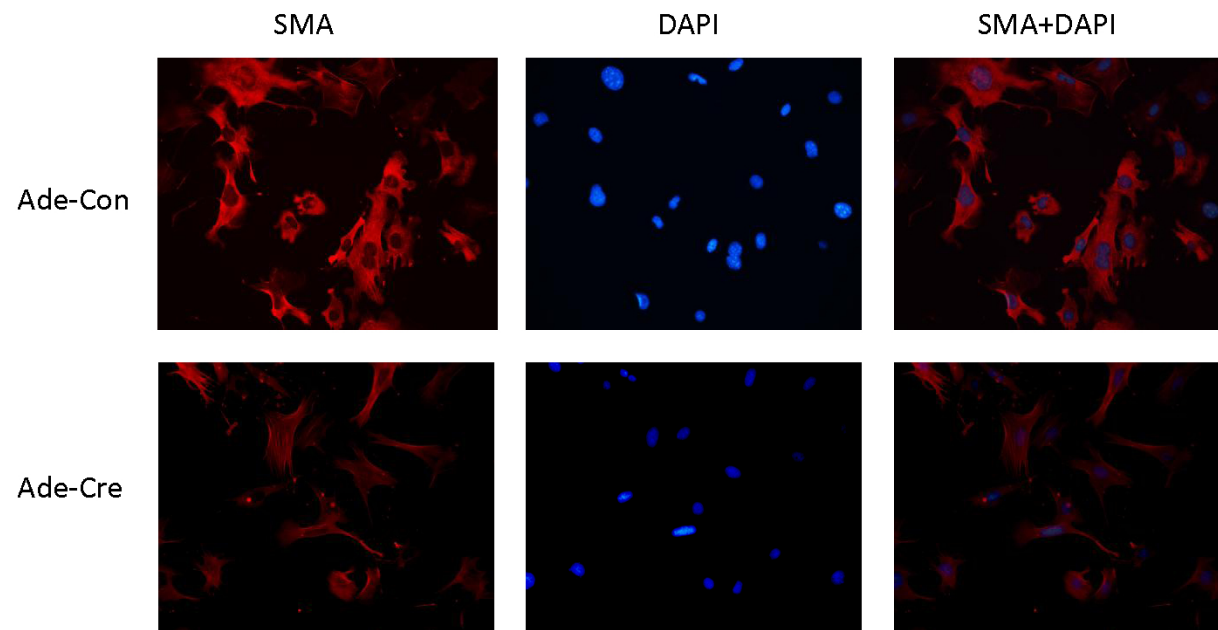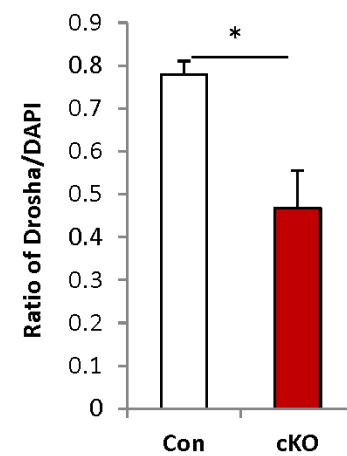

Supplement: Figure S2 — Immunostaining of SMA in KO VSMCs. The expressions of SMA in Drosha KO and control VSMCs generated using Ade-con and Ade-Cre were examined by immunostaining using SMA antibody; the significant differences were analyzed from four separate experiments by quantifying the fluorescent intensity in the Drosha KO VSMCs compared with controls (*P<0.05). (PDF) [file pone.0060888.s002.pdf]

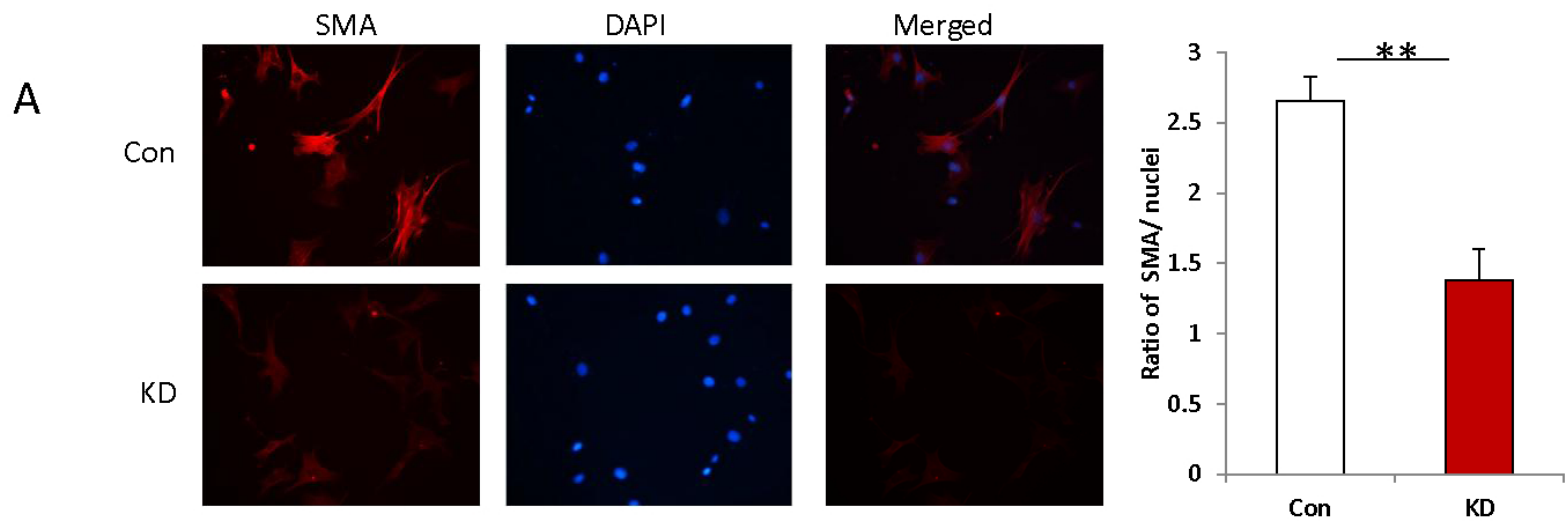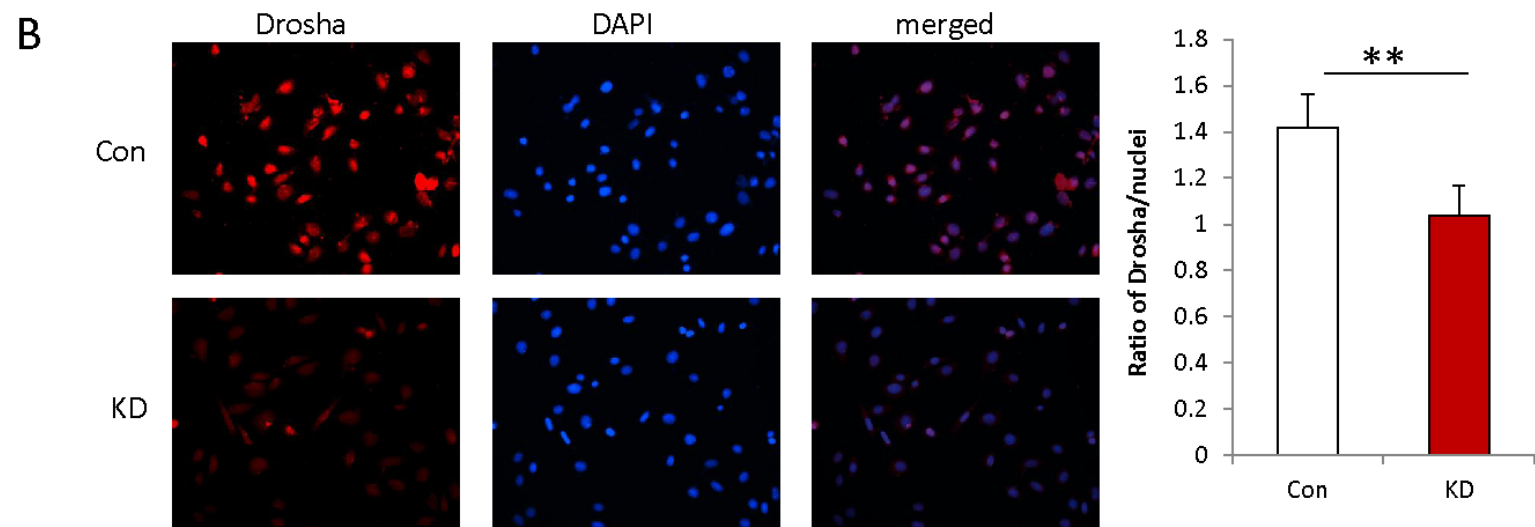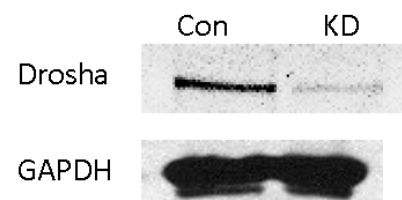

Supplement: Figure S3 — Immunostaining of SMA and Drosha in KD VSMCs. A. The expressions of SMA in Drosha KD and control VSMCs generated using retroviral shRNA vector were examined by immunostaining using SMA antibody; the significant differences were analyzed from three separate experiments by quantifying the fluorescent intensity in the Drosha KO VSMCs compared with controls (**P<0.01). B. The expressions of Drosha in KD and control VSMCs were examined using immunostaining and Western blot. Three separate experiments were performed, and significances were analyzed (**P<0.01). (PDF) [file pone.0060888.s003.pdf]

Figure S4

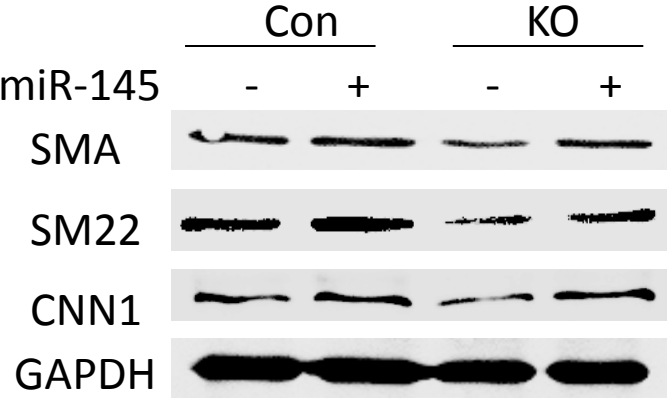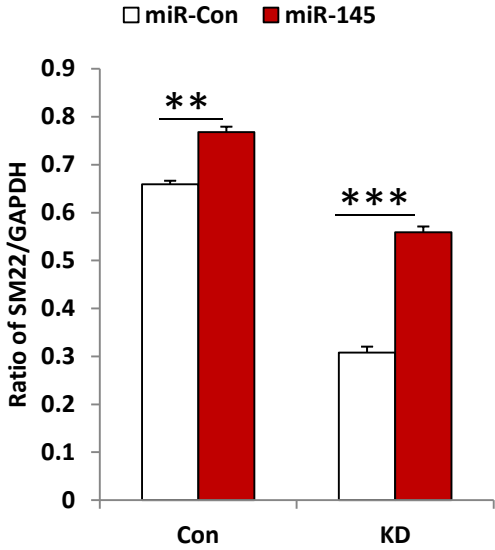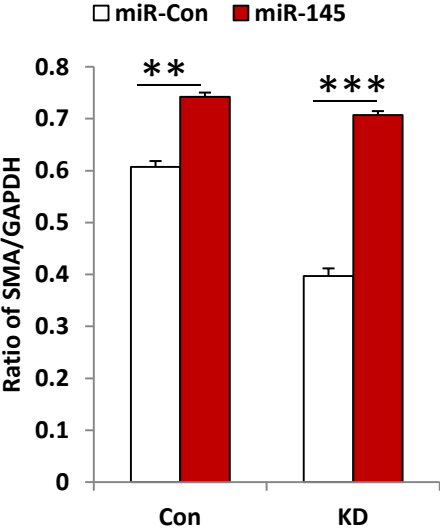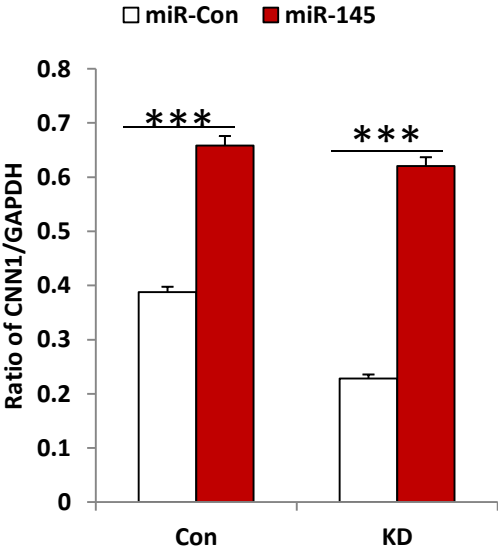

Supplement: Figure S4 — Rescue VSMC marker gene expressions in Drosha KD VSMCs. Drosha KD and control VSMCs were transduced using miR-145 and control lentiviral vector, respectively. The expressions of VSMC marker genes including SMA, SM22, and CNN1 were detected using Western blot. The significant differences were determined by measuring band intensity and calculated from three separate experiments (*P<0.05). (PDF) [file pone.0060888.s004.pdf]

Figure.S5

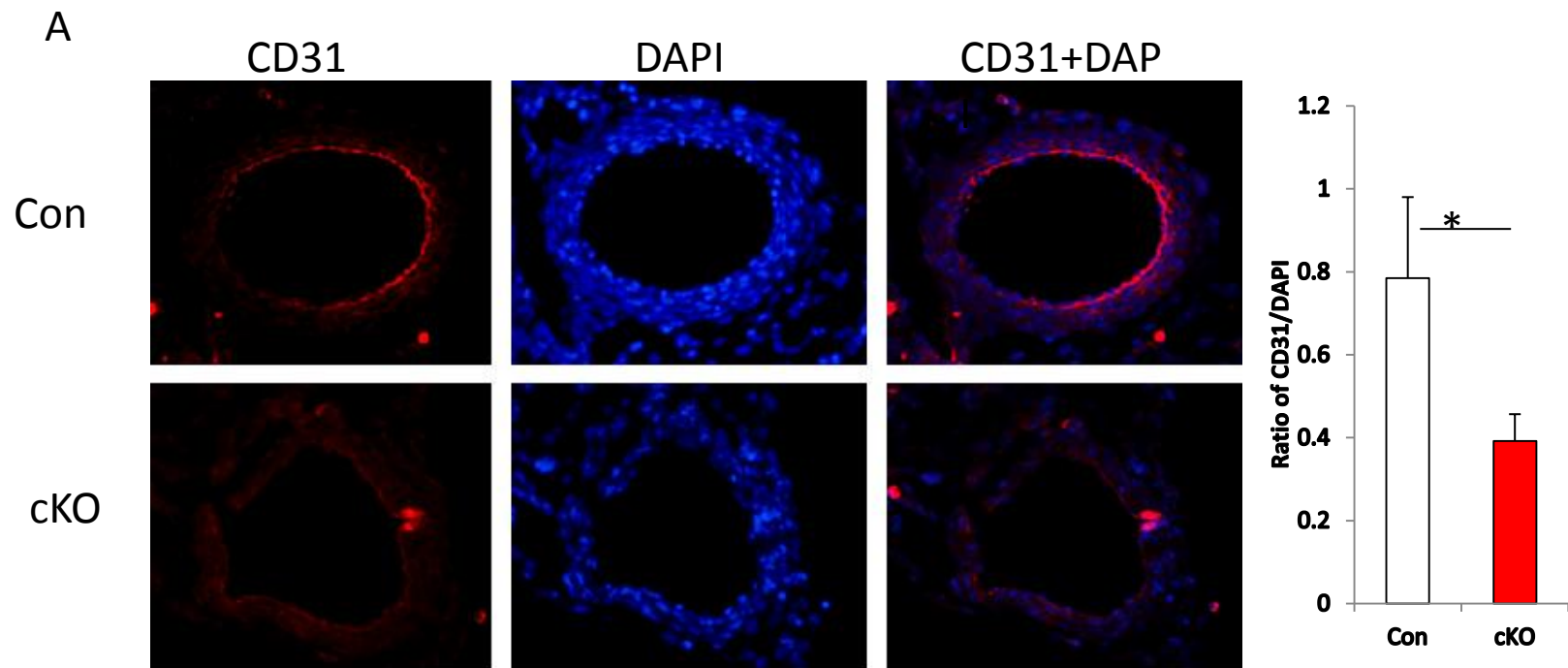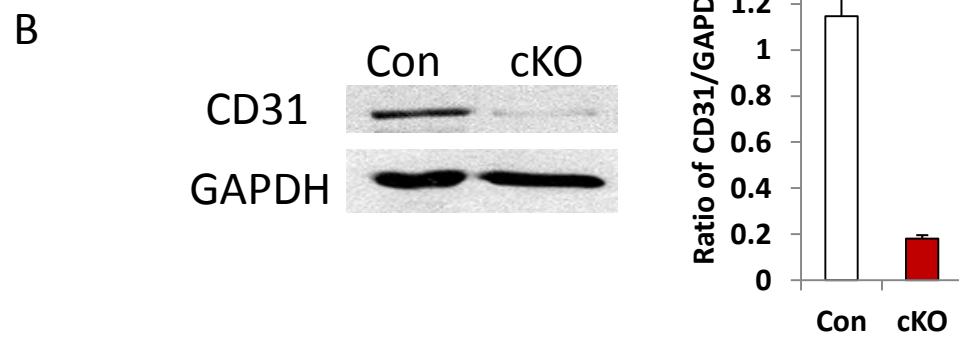

Supplement: Figure S5 — Immunostaining and immunobloting of CD31 in thoracic aorta. A. The thoracic aorta of Drosha cKO and control embryos at E14.5 were stained with endothelial cell marker CD31 (*P<0.05). B. The expression of endothelial cell marker CD31 was examined in umbilical arteries of E14.5 in Drosha cKO and control embryos by Western blot (**P<0.01) (PDF) [file pone.0060888.s005.pdf]

Figure S6

A

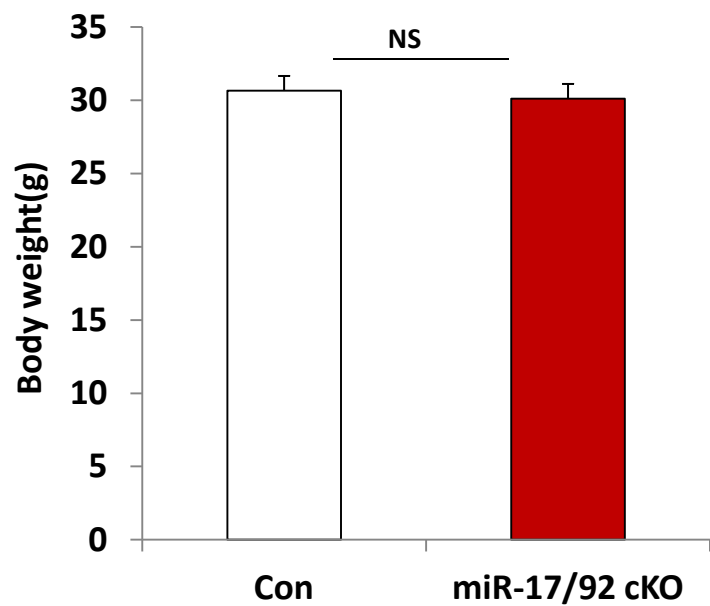

B

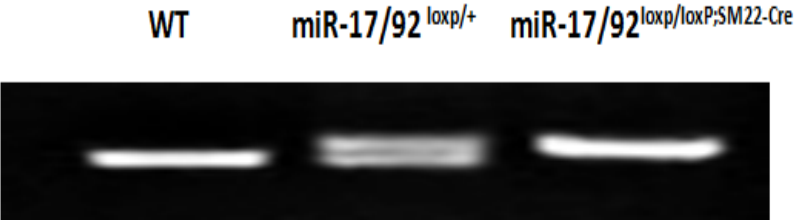

Supplement: Figure S6 — miR-17/92 VSMC-specific KO mice are developmentally normal. A. The bodyweight of 1-month-old miR-17/92 VSMC cKO mice were compared with that of controls (n = 6, NS: no significance ). B. Three different genotypes of miR-17/92 cKO mice were detected by PCR. (PDF) [file pone.0060888.s006.pdf]
