# Supplementary material for: miRNA Biogenesis Enzyme Drosha Is Required for Vascular Smooth Muscle Cell Survival
Source: PLoS One. 2013 Apr 18;8(4):e60888. doi: 10.1371/journal.pone.0060888 (PMC3630177; doi:10.1371/journal.pone.0060888)
Supplement: Table S1 — Phenotypic comparison of VSMC-specific Drosha, DGCR8, and Dicer cKO mice. Phenotypic differences among Drosha, DGCR8, and Dicer cKO were compared to controls and summarized in Table 1. (PDF) [file pone.0060888.s007.pdf]

Table S1 Phenotypic comparsion of VSMC specific Drosha, DGCR8 and Dicer cKO mice

	Drosha	DGCR8	Dicer
Lethality time	E14.5	E11.5~12.5	E15.5~16.5
Growth delay	No	Yes	Yes
Liver hemorrhage	Yes	Yes	Yes
miR-143/145	Downregulated	Downregulated	Downregulated

Table S2: miRNA expression profiles in the umbilical arteries of Drosha cKO and control

ProbeSet Name	cKO_(miRNA-1_0)	p-value (cKO_(miRNA-1_0))	Control_(miRNA-1_0)	p-value (Control_(miRNA-1_0))
mmu-let-7g_st	176.0992	0.00552743	108.186	0.008013108
mmu-let-7i_st	1010.36	6.88E-06	760.2106	1.17E-05
mmu-miR-15b_st	365.4714	0.000344788	256.6574	0.001205841
mmu-miR-23b_st	2022.741	2.22E-06	2218.135	2.05E-08
mmu-miR-27b_st	191.2281	0.007744181	368.1843	0.000274656
mmu-miR-30a_st	173.414	0.007182509	415.7555	6.81E-05
mmu-miR-30b_st	148.3776	0.007843355	303.7594	0.000730234
mmu-miR-99a_st	276.2535	0.002874706	697.3185	5.60E-05
mmu-miR-99b_st	2708.069	2.05E-08	3522.612	2.05E-08
mmu-miR-125a-5p_st	1612.755	1.04E-06	1299.439	3.83E-08
mmu-miR-125b-5p_st	1675.037	3.83E-08	1952.411	1.04E-06
mmu-miR-126-3p_st	704.0233	9.69E-05	944.9069	7.27E-07
mmu-miR-127_st	5350.058	2.05E-08	7316.074	2.05E-08
mmu-miR-130a_st	1028.398	7.73E-06	2556.891	2.05E-08
mmu-miR-134_st	1808.61	7.27E-07	1362.063	9.37E-07
mmu-miR-138_st	153.2999	0.02328095	389.842	0.000920161
mmu-miR-141_st	98.30212	0.03511543	317.4154	0.000416083
mmu-miR-145_st	1801.537	1.04E-06	4195.622	2.05E-08
mmu-miR-151-5p_st	514.0986	7.33E-05	515.8688	0.000140257
mmu-miR-151-3p_st	120.7621	0.02478577	109.9179	0.02201726
mmu-miR-152_st	1135.005	5.00E-05	1469.891	2.47E-07
mmu-miR-154_st	134.6969	0.04270166	254.9351	0.003405793
mmu-miR-181a_st	514.48	7.33E-05	977.9589	1.17E-05
mmu-miR-182_st	1436.464	7.27E-07	1286.858	2.05E-08
mmu-miR-183_st	119.537	0.02550048	111.6547	0.01621607
mmu-miR-185_st	1104.242	5.91E-06	696.0083	4.48E-05
mmu-miR-24_st	3954.94	2.05E-08	3916.56	2.05E-08
mmu-miR-191_st	3036.339	2.47E-07	2497.278	2.05E-08
mmu-miR-194_st	1949.25	2.80E-06	1714.731	3.83E-08
mmu-miR-199a-5p_st	625.414	4.21E-05	1890.727	2.05E-08
mmu-miR-199a-3p_st	1434.139	2.05E-08	1238.452	1.04E-06

mmu-miR-200b_st	208.6181	0.004736582	162.1406	0.006250085
mmu-miR-203_st	122.2113	0.01377449	325.602	0.000333534
mmu-miR-143_st	1178.853	5.00E-05	3386.285	2.05E-08
mmu-miR-290-5p_st	623.6066	0.000184229	454.0496	6.90E-05
mmu-miR-292-5p_st	303.058	0.002124524	147.3061	0.0100027
mmu-miR-292-3p_st	862.7097	1.93E-05	399.0946	0.000124184
mmu-miR-293_st	383.4983	0.000317094	313.4342	0.001079096
mmu-miR-296-3p_st	142.2623	0.02111623	615.8486	5.30E-06
mmu-miR-298_st	898.5653	2.96E-05	820.9673	3.50E-07
mmu-miR-299_st	159.852	0.00633913	322.4022	0.001154355
mmu-miR-300_st	575.5774	6.76E-05	1086.295	8.56E-06
mmu-let-7d_st	2384.941	2.05E-08	2530.074	2.05E-08
mmu-miR-106a_st	868.4946	9.61E-06	1243.282	4.23E-06
mmu-miR-106b_st	619.6028	2.21E-05	1363.812	1.04E-06
mmu-miR-130b_st	423.6593	0.000521098	1628.115	1.33E-07
mmu-miR-19b_st	761.619	6.82E-06	1503.612	7.27E-07
mmu-miR-30c_st	424.353	0.000337986	540.5021	1.16E-05
mmu-miR-30d_st	331.5821	0.000527039	297.0122	0.001036446
mmu-miR-192_st	512.6977	0.000254757	516.5609	1.65E-05
mmu-miR-200a_st	138.6504	0.01235516	292.858	0.000683212
mmu-let-7a_st	1485.854	6.02E-06	851.3195	5.08E-07
mmu-let-7b_st	1028.615	6.76E-05	1146.523	2.47E-07
mmu-let-7c_st	2462.541	2.05E-08	2397.673	2.05E-08
mmu-let-7e_st	3434.265	2.05E-08	3430.966	2.05E-08
mmu-miR-16_st	3501.497	2.05E-08	4067.921	2.05E-08
mmu-miR-18a_st	467.0322	0.000377674	1761.703	7.15E-08
mmu-miR-20a_st	1518.645	2.05E-08	2584.058	2.05E-08
mmu-miR-22_st	1564.753	6.24E-06	1364.305	1.33E-07
mmu-miR-23a_st	2977.627	7.15E-08	1622.544	1.33E-07
mmu-miR-26a_st	5343.014	2.05E-08	4759.583	2.05E-08
mmu-miR-27a_st	478.8503	7.33E-05	737.3399	2.00E-05
mmu-miR-31_st	1017.54	7.31E-06	1222.366	3.83E-08
mmu-miR-92a_st	898.1454	1.00E-05	978.5997	1.91E-07

mmu-miR-93_st	1421.174	1.04E-06	2119.031	2.05E-08
mmu-miR-34a_st	139.0831	0.001535555	469.9535	0.00034695
mmu-miR-103_st	4095.697	2.05E-08	4864.242	2.05E-08
mmu-miR-324-5p_st	402.6872	0.000193812	607.2008	5.42E-05
mmu-miR-329_st	574.9465	6.42E-05	316.3927	0.00073442
mmu-miR-337-5p_st	747.5455	1.61E-05	856.6932	7.51E-07
mmu-miR-341_st	55.53144	0.02865593	192.4959	0.00368462
mmu-miR-342-5p_st	107.2879	0.04094442	81.7449	0.03521013
mmu-miR-342-3p_st	898.1776	7.36E-06	1010.131	2.47E-07
mmu-miR-345-5p_st	79.5938	0.03964012	291.0197	0.002388124
mmu-miR-351_st	1474.997	2.93E-08	1939.38	2.93E-08
mmu-miR-107_st	3472.073	2.05E-08	3422.32	2.05E-08
mmu-miR-17_st	2474.406	2.05E-08	3905.973	2.05E-08
mmu-miR-25_st	255.415	0.001620762	322.8605	0.001995062
mmu-miR-28_st	186.9097	0.008141229	228.7461	0.002471312
mmu-miR-100_st	214.3012	0.002836205	346.8808	0.000786766
mmu-miR-139-5p_st	95.91883	0.04319737	87.81985	0.05308421
mmu-miR-200c_st	3276.96	2.05E-08	2323.351	2.05E-08
mmu-miR-210_st	1013.254	4.57E-07	1245.449	2.47E-07
mmu-miR-214_st	3163.283	2.05E-08	3715.81	2.05E-08
mmu-miR-320_st	612.557	5.98E-05	398.537	0.000292324
mmu-miR-199b_st	1387.567	2.05E-08	1095.811	1.73E-06
mmu-miR-181b_st	461.2577	0.00010566	1142.41	7.31E-06
mmu-miR-181c_st	80.37236	0.01256541	161.8151	0.000861621
mmu-miR-361_st	667.2245	0.000151122	645.0219	8.26E-06
mmu-miR-362-5p_st	151.5156	0.005778691	290.016	0.001683965
mmu-miR-376a_st	369.6392	0.00033502	418.0526	0.000210077
mmu-miR-378_st	394.854	0.000329566	527.1481	0.000140257
mmu-miR-379_st	2189.631	2.22E-06	2352.588	2.05E-08
mmu-miR-380-5p_st	187.2351	0.002387535	148.0005	0.01516094
mmu-miR-382_st	1771.454	3.83E-08	1011.602	1.17E-05
mmu-miR-335-5p_st	195.2896	0.002027167	95.47771	0.02941277
mmu-miR-409-5p_st	543.0111	6.65E-05	593.4643	8.62E-05

mmu-miR-409-3p_st	1748.091	1.04E-06	1366.791	2.05E-08
mmu-miR-410_st	120.6222	0.02190318	247.6609	0.001614586
mmu-miR-376b_st	662.2132	4.68E-05	963.5573	1.03E-07
mmu-miR-411_st	1099.795	8.06E-06	966.5773	7.27E-07
mmu-miR-370_st	279.4746	0.003349749	408.2409	6.54E-05
mmu-miR-425_st	280.6487	0.001040226	377.0183	0.000382472
mmu-miR-431_st	1899.862	1.04E-06	2178.567	2.05E-08
mmu-miR-433_st	1287.999	1.04E-06	1124.765	7.15E-08
mmu-miR-434-5p_st	136.725	0.0264323	476.4447	0.000259898
mmu-miR-434-3p_st	812.6711	0.000107446	497.3513	4.47E-05
mmu-miR-429_st	347.1818	0.000395545	393.5388	0.000234836
mmu-miR-466a-3p_st	371.8141	0.000448597	115.4837	0.01605521
mmu-miR-467a_st	580.8785	0.000177952	426.7704	0.00015953
mmu-miR-470_st	98.35048	0.03448163	76.37605	0.04389229
mmu-miR-532-5p_st	455.8232	0.000134007	536.9868	1.69E-05
mmu-miR-532-3p_st	142.0254	0.01458428	92.83827	0.03002472
mmu-miR-483_st	146.987	0.02089787	224.1222	0.000547947
mmu-miR-485_st	176.4058	0.00916348	252.5184	0.002108217
mmu-miR-486_st	279.4654	0.001812119	182.983	0.002789673
mmu-miR-540-3p_st	229.1801	0.004993885	282.9478	0.000908451
mmu-miR-543_st	369.9621	0.000960249	229.024	0.002403343
mmu-miR-541_st	3725.657	2.05E-08	3198.71	2.05E-08
mmu-miR-542-5p_st	125.8756	0.02366951	189.4741	0.003193134
mmu-miR-494_st	1207.161	3.23E-05	954.7923	5.08E-07
mmu-miR-487b_st	619.5049	0.002694933	1063.641	5.08E-07
mmu-miR-20b_st	155.0703	0.006899295	523.2933	9.67E-05
mmu-miR-503_st	1631.03	1.04E-06	2705.819	2.05E-08
mmu-miR-1224_st	415.3479	0.000192724	219.3755	0.00201043
mmu-miR-301b_st	96.68697	0.02161252	171.29	0.00586197
mmu-miR-744_st	156.9418	0.009162338	234.088	0.002340553
mmu-miR-374_st	198.7983	0.02024719	44.43887	0.01663768
mmu-miR-668_st	201.9133	0.0029128	407.2525	0.000131787
mmu-miR-665_st	521.3515	0.000374237	1153.977	5.13E-06

mmu-miR-667_st	378.5799	0.00017161	329.079	3.83E-05
mmu-miR-762_st	1043.894	4.81E-05	224.7284	0.002088536
mmu-miR-674_st	313.6601	0.000182608	708.9845	1.08E-05
mmu-miR-423-5p_st	147.6743	0.02079581	222.5164	0.003402603
mmu-miR-423-3p_st	108.8306	0.04076628	188.6387	0.004829697
mmu-miR-495_st	273.7988	0.00181954	178.0226	0.008855181
mmu-miR-685_st	119.6766	0.03019315	141.9177	0.01247926
mmu-miR-690_st	5203.148	2.05E-08	12088.44	2.05E-08
mmu-miR-691_st	69.42973	0.04862881	68.3583	0.05626863
mmu-miR-669c_st	449.0959	0.000167846	175.5547	0.004695422
mmu-miR-720_st	1521.539	1.04E-06	1391.025	2.05E-08
mmu-miR-455_st	217.1344	0.003855135	387.7581	0.000432971
mmu-miR-700_st	149.298	0.001293773	152.427	0.02286998
mmu-miR-705_st	345.2979	0.000664432	217.9762	0.002838516
mmu-miR-706_st	113.0049	0.02433994	106.501	0.01874034
mmu-miR-708_st	163.3116	0.01267272	582.5062	9.34E-05
mmu-miR-709_st	12205.73	2.05E-08	12117.14	2.05E-08
mmu-miR-711_st	125.522	0.003255878	67.23685	0.01802134
mmu-miR-714_st	127.6634	0.02489339	133.8224	0.01856779
mmu-miR-500_st	372.0608	0.000374889	430.7616	0.00026246
mmu-miR-501-3p_st	229.4004	0.006309438	252.439	0.000145025
mmu-miR-721_st	85.11152	0.04064561	63.59674	0.02269715
mmu-miR-652_st	373.7676	0.000599902	776.321	1.21E-06
mmu-miR-615-3p_st	169.1551	0.01034936	279.8497	0.000926106
mmu-miR-805_st	138.0535	0.01038346	124.6132	0.01245285
mmu-miR-743a_st	118.519	0.02379829	163.0912	0.01020397
mmu-miR-181d_st	130.6767	0.009649343	392.4883	0.000377493
mmu-miR-193b_st	119.5665	0.0323408	349.024	0.000743636
mmu-miR-421_st	141.2342	0.01857322	129.8846	0.002458914
mmu-miR-466b-3p_st	228.6653	0.000259514	75.79465	0.004933881
mmu-miR-466b-3-3p_st	181.6	0.006064159	63.50697	0.01906401
mmu-miR-466c-3p_st	358.9465	0.001309796	195.3029	0.000633791
mmu-miR-466e-3p_st	306.9953	0.001785924	207.7937	0.000684763

mmu-miR-466f-3p_st	374.0187	0.000267811	409.0064	0.000210077
mmu-miR-466g_st	319.1982	0.000298034	173.231	0.00605024
mmu-miR-574-5p_st	168.899	0.009371428	282.0978	0.001338107
mmu-miR-574-3p_st	405.0378	0.00132885	338.9185	0.001162
mmu-miR-669k_st	92.06111	0.05527418	145.4735	0.01898616
mmu-miR-466i_st	269.6688	0.003132826	487.3692	3.81E-05
mmu-miR-1187_st	130.0574	0.02819728	175.2188	0.007413736
mmu-miR-467f_st	178.4973	0.009894461	197.8085	0.001718257
mmu-miR-466j_st	85.69039	0.03647212	61.61609	0.04277109
mmu-miR-1195_st	686.7464	3.29E-06	405.6268	0.000235709
mmu-miR-1196_st	1162.916	5.00E-05	216.5091	0.003531655
mmu-miR-99b-star_st	158.3648	0.01335267	277.8055	0.001239205
mmu-miR-127-star_st	262.5748	0.001810704	444.5723	0.000203406
mmu-miR-133a-star_st	131.8002	0.004082429	51.63229	0.03811923
mmu-miR-140-star_st	526.92	0.000182424	807.8068	1.38E-06
mmu-miR-150-star_st	152.771	0.006581948	15.90168	0.03193578
mmu-miR-193-star_st	77.78293	0.03497078	227.0359	0.003171266
mmu-miR-200b-star_st	134.5147	0.0188619	94.2515	0.04025601
mmu-miR-299-star_st	121.646	0.0178301	191.1412	0.004914902
mmu-miR-106b-star_st	227.666	0.002870493	305.3827	0.000705435
mmu-miR-15a-star_st	138.2123	0.02354804	174.6201	0.02182049
mmu-miR-31-star_st	96.13125	0.01492252	84.18771	0.01215605
mmu-miR-322-star_st	763.2585	9.83E-05	958.18	3.54E-07
mmu-miR-17-star_st	287.3819	0.00271912	464.9563	0.000198981
mmu-miR-28-star_st	131.6655	0.03680382	158.8175	0.008718664
mmu-miR-199b-star_st	122.4032	0.03223754	238.7198	0.00236388
mmu-miR-7a-star_st	108.6409	0.01636171	62.78576	0.05925293
mmu-miR-411-star_st	177.104	0.004445613	185.1082	0.002220236
mmu-miR-467a-star_st	340.381	0.000729368	117.8945	0.02738829
mmu-miR-485-star_st	417.9315	0.000313934	333.6454	0.000532186
mmu-miR-467b-star_st	182.3038	0.005675805	167.0405	0.006992011
mmu-miR-881-star_st	100.5125	0.02873719	96.03547	0.02589986
mmu-miR-467d-star_st	252.2046	0.001830111	134.7229	0.02112495

mmu-miR-467e-star_st	110.6878	0.03252307	124.8851	0.004765702
