# Supplementary material for: miRNA Biogenesis Enzyme Drosha Is Required for Vascular Smooth Muscle Cell Survival
Source: PLoS One. 2013 Apr 18;8(4):e60888. doi: 10.1371/journal.pone.0060888 (PMC3630177; doi:10.1371/journal.pone.0060888)
Supplement: Table S2 — miRNA expression profiles in the umbilical arteries of Drosha cKO and control embryos. miRNA array was performed using RNA samples extracted from umbilical arteries of E13.5 Drosha cKO and control embryos (n = 6). miRNA expressions and P values for both Drosha cKO and controls. (PDF) [file pone.0060888.s008.pdf]

Table S2: miRNA expression profiles in the umbilical arteries of Drosha cKO and control

ProbeSet Name	cKO_(miRNA-1_0)	p-value (cKO_(miRNA-1_0))	Control_(miRNA-1_0)	p-value (Control_(miRNA-1_0))
mmu-let-7g_st	176.0992	0.00552743	108.186	0.008013108
mmu-let-7i_st	1010.36	6.88E-06	760.2106	1.17E-05
mmu-miR-15b_st	365.4714	0.000344788	256.6574	0.001205841
mmu-miR-23b_st	2022.741	2.22E-06	2218.135	2.05E-08
mmu-miR-27b_st	191.2281	0.007744181	368.1843	0.000274656
mmu-miR-30a_st	173.414	0.007182509	415.7555	6.81E-05
mmu-miR-30b_st	148.3776	0.007843355	303.7594	0.000730234
mmu-miR-99a_st	276.2535	0.002874706	697.3185	5.60E-05
mmu-miR-99b_st	2708.069	2.05E-08	3522.612	2.05E-08
mmu-miR-125a-5p_st	1612.755	1.04E-06	1299.439	3.83E-08
mmu-miR-125b-5p_st	1675.037	3.83E-08	1952.411	1.04E-06
mmu-miR-126-3p_st	704.0233	9.69E-05	944.9069	7.27E-07
mmu-miR-127_st	5350.058	2.05E-08	7316.074	2.05E-08
mmu-miR-130a_st	1028.398	7.73E-06	2556.891	2.05E-08
mmu-miR-134_st	1808.61	7.27E-07	1362.063	9.37E-07
mmu-miR-138_st	153.2999	0.02328095	389.842	0.000920161
mmu-miR-141_st	98.30212	0.03511543	317.4154	0.000416083
mmu-miR-145_st	1801.537	1.04E-06	4195.622	2.05E-08
mmu-miR-151-5p_st	514.0986	7.33E-05	515.8688	0.000140257
mmu-miR-151-3p_st	120.7621	0.02478577	109.9179	0.02201726
mmu-miR-152_st	1135.005	5.00E-05	1469.891	2.47E-07
mmu-miR-154_st	134.6969	0.04270166	254.9351	0.003405793
mmu-miR-181a_st	514.48	7.33E-05	977.9589	1.17E-05
mmu-miR-182_st	1436.464	7.27E-07	1286.858	2.05E-08
mmu-miR-183_st	119.537	0.02550048	111.6547	0.01621607
mmu-miR-185_st	1104.242	5.91E-06	696.0083	4.48E-05
mmu-miR-24_st	3954.94	2.05E-08	3916.56	2.05E-08
mmu-miR-191_st	3036.339	2.47E-07	2497.278	2.05E-08
mmu-miR-194_st	1949.25	2.80E-06	1714.731	3.83E-08
mmu-miR-199a-5p_st	625.414	4.21E-05	1890.727	2.05E-08
mmu-miR-199a-3p_st	1434.139	2.05E-08	1238.452	1.04E-06

mmu-miR-200b_st	208.6181	0.004736582	162.1406	0.006250085
mmu-miR-203_st	122.2113	0.01377449	325.602	0.000333534
mmu-miR-143_st	1178.853	5.00E-05	3386.285	2.05E-08
mmu-miR-290-5p_st	623.6066	0.000184229	454.0496	6.90E-05
mmu-miR-292-5p_st	303.058	0.002124524	147.3061	0.0100027
mmu-miR-292-3p_st	862.7097	1.93E-05	399.0946	0.000124184
mmu-miR-293_st	383.4983	0.000317094	313.4342	0.001079096
mmu-miR-296-3p_st	142.2623	0.02111623	615.8486	5.30E-06
mmu-miR-298_st	898.5653	2.96E-05	820.9673	3.50E-07
mmu-miR-299_st	159.852	0.00633913	322.4022	0.001154355
mmu-miR-300_st	575.5774	6.76E-05	1086.295	8.56E-06
mmu-let-7d_st	2384.941	2.05E-08	2530.074	2.05E-08
mmu-miR-106a_st	868.4946	9.61E-06	1243.282	4.23E-06
mmu-miR-106b_st	619.6028	2.21E-05	1363.812	1.04E-06
mmu-miR-130b_st	423.6593	0.000521098	1628.115	1.33E-07
mmu-miR-19b_st	761.619	6.82E-06	1503.612	7.27E-07
mmu-miR-30c_st	424.353	0.000337986	540.5021	1.16E-05
mmu-miR-30d_st	331.5821	0.000527039	297.0122	0.001036446
mmu-miR-192_st	512.6977	0.000254757	516.5609	1.65E-05
mmu-miR-200a_st	138.6504	0.01235516	292.858	0.000683212
mmu-let-7a_st	1485.854	6.02E-06	851.3195	5.08E-07
mmu-let-7b_st	1028.615	6.76E-05	1146.523	2.47E-07
mmu-let-7c_st	2462.541	2.05E-08	2397.673	2.05E-08
mmu-let-7e_st	3434.265	2.05E-08	3430.966	2.05E-08
mmu-miR-16_st	3501.497	2.05E-08	4067.921	2.05E-08
mmu-miR-18a_st	467.0322	0.000377674	1761.703	7.15E-08
mmu-miR-20a_st	1518.645	2.05E-08	2584.058	2.05E-08
mmu-miR-22_st	1564.753	6.24E-06	1364.305	1.33E-07
mmu-miR-23a_st	2977.627	7.15E-08	1622.544	1.33E-07
mmu-miR-26a_st	5343.014	2.05E-08	4759.583	2.05E-08
mmu-miR-27a_st	478.8503	7.33E-05	737.3399	2.00E-05
mmu-miR-31_st	1017.54	7.31E-06	1222.366	3.83E-08
mmu-miR-92a_st	898.1454	1.00E-05	978.5997	1.91E-07

mmu-miR-93_st	1421.174	1.04E-06	2119.031	2.05E-08
mmu-miR-34a_st	139.0831	0.001535555	469.9535	0.00034695
mmu-miR-103_st	4095.697	2.05E-08	4864.242	2.05E-08
mmu-miR-324-5p_st	402.6872	0.000193812	607.2008	5.42E-05
mmu-miR-329_st	574.9465	6.42E-05	316.3927	0.00073442
mmu-miR-337-5p_st	747.5455	1.61E-05	856.6932	7.51E-07
mmu-miR-341_st	55.53144	0.02865593	192.4959	0.00368462
mmu-miR-342-5p_st	107.2879	0.04094442	81.7449	0.03521013
mmu-miR-342-3p_st	898.1776	7.36E-06	1010.131	2.47E-07
mmu-miR-345-5p_st	79.5938	0.03964012	291.0197	0.002388124
mmu-miR-351_st	1474.997	2.93E-08	1939.38	2.93E-08
mmu-miR-107_st	3472.073	2.05E-08	3422.32	2.05E-08
mmu-miR-17_st	2474.406	2.05E-08	3905.973	2.05E-08
mmu-miR-25_st	255.415	0.001620762	322.8605	0.001995062
mmu-miR-28_st	186.9097	0.008141229	228.7461	0.002471312
mmu-miR-100_st	214.3012	0.002836205	346.8808	0.000786766
mmu-miR-139-5p_st	95.91883	0.04319737	87.81985	0.05308421
mmu-miR-200c_st	3276.96	2.05E-08	2323.351	2.05E-08
mmu-miR-210_st	1013.254	4.57E-07	1245.449	2.47E-07
mmu-miR-214_st	3163.283	2.05E-08	3715.81	2.05E-08
mmu-miR-320_st	612.557	5.98E-05	398.537	0.000292324
mmu-miR-199b_st	1387.567	2.05E-08	1095.811	1.73E-06
mmu-miR-181b_st	461.2577	0.00010566	1142.41	7.31E-06
mmu-miR-181c_st	80.37236	0.01256541	161.8151	0.000861621
mmu-miR-361_st	667.2245	0.000151122	645.0219	8.26E-06
mmu-miR-362-5p_st	151.5156	0.005778691	290.016	0.001683965
mmu-miR-376a_st	369.6392	0.00033502	418.0526	0.000210077
mmu-miR-378_st	394.854	0.000329566	527.1481	0.000140257
mmu-miR-379_st	2189.631	2.22E-06	2352.588	2.05E-08
mmu-miR-380-5p_st	187.2351	0.002387535	148.0005	0.01516094
mmu-miR-382_st	1771.454	3.83E-08	1011.602	1.17E-05
mmu-miR-335-5p_st	195.2896	0.002027167	95.47771	0.02941277
mmu-miR-409-5p_st	543.0111	6.65E-05	593.4643	8.62E-05

mmu-miR-409-3p_st	1748.091	1.04E-06	1366.791	2.05E-08
mmu-miR-410_st	120.6222	0.02190318	247.6609	0.001614586
mmu-miR-376b_st	662.2132	4.68E-05	963.5573	1.03E-07
mmu-miR-411_st	1099.795	8.06E-06	966.5773	7.27E-07
mmu-miR-370_st	279.4746	0.003349749	408.2409	6.54E-05
mmu-miR-425_st	280.6487	0.001040226	377.0183	0.000382472
mmu-miR-431_st	1899.862	1.04E-06	2178.567	2.05E-08
mmu-miR-433_st	1287.999	1.04E-06	1124.765	7.15E-08
mmu-miR-434-5p_st	136.725	0.0264323	476.4447	0.000259898
mmu-miR-434-3p_st	812.6711	0.000107446	497.3513	4.47E-05
mmu-miR-429_st	347.1818	0.000395545	393.5388	0.000234836
mmu-miR-466a-3p_st	371.8141	0.000448597	115.4837	0.01605521
mmu-miR-467a_st	580.8785	0.000177952	426.7704	0.00015953
mmu-miR-470_st	98.35048	0.03448163	76.37605	0.04389229
mmu-miR-532-5p_st	455.8232	0.000134007	536.9868	1.69E-05
mmu-miR-532-3p_st	142.0254	0.01458428	92.83827	0.03002472
mmu-miR-483_st	146.987	0.02089787	224.1222	0.000547947
mmu-miR-485_st	176.4058	0.00916348	252.5184	0.002108217
mmu-miR-486_st	279.4654	0.001812119	182.983	0.002789673
mmu-miR-540-3p_st	229.1801	0.004993885	282.9478	0.000908451
mmu-miR-543_st	369.9621	0.000960249	229.024	0.002403343
mmu-miR-541_st	3725.657	2.05E-08	3198.71	2.05E-08
mmu-miR-542-5p_st	125.8756	0.02366951	189.4741	0.003193134
mmu-miR-494_st	1207.161	3.23E-05	954.7923	5.08E-07
mmu-miR-487b_st	619.5049	0.002694933	1063.641	5.08E-07
mmu-miR-20b_st	155.0703	0.006899295	523.2933	9.67E-05
mmu-miR-503_st	1631.03	1.04E-06	2705.819	2.05E-08
mmu-miR-1224_st	415.3479	0.000192724	219.3755	0.00201043
mmu-miR-301b_st	96.68697	0.02161252	171.29	0.00586197
mmu-miR-744_st	156.9418	0.009162338	234.088	0.002340553
mmu-miR-374_st	198.7983	0.02024719	44.43887	0.01663768
mmu-miR-668_st	201.9133	0.0029128	407.2525	0.000131787
mmu-miR-665_st	521.3515	0.000374237	1153.977	5.13E-06

mmu-miR-667_st	378.5799	0.00017161	329.079	3.83E-05
mmu-miR-762_st	1043.894	4.81E-05	224.7284	0.002088536
mmu-miR-674_st	313.6601	0.000182608	708.9845	1.08E-05
mmu-miR-423-5p_st	147.6743	0.02079581	222.5164	0.003402603
mmu-miR-423-3p_st	108.8306	0.04076628	188.6387	0.004829697
mmu-miR-495_st	273.7988	0.00181954	178.0226	0.008855181
mmu-miR-685_st	119.6766	0.03019315	141.9177	0.01247926
mmu-miR-690_st	5203.148	2.05E-08	12088.44	2.05E-08
mmu-miR-691_st	69.42973	0.04862881	68.3583	0.05626863
mmu-miR-669c_st	449.0959	0.000167846	175.5547	0.004695422
mmu-miR-720_st	1521.539	1.04E-06	1391.025	2.05E-08
mmu-miR-455_st	217.1344	0.003855135	387.7581	0.000432971
mmu-miR-700_st	149.298	0.001293773	152.427	0.02286998
mmu-miR-705_st	345.2979	0.000664432	217.9762	0.002838516
mmu-miR-706_st	113.0049	0.02433994	106.501	0.01874034
mmu-miR-708_st	163.3116	0.01267272	582.5062	9.34E-05
mmu-miR-709_st	12205.73	2.05E-08	12117.14	2.05E-08
mmu-miR-711_st	125.522	0.003255878	67.23685	0.01802134
mmu-miR-714_st	127.6634	0.02489339	133.8224	0.01856779
mmu-miR-500_st	372.0608	0.000374889	430.7616	0.00026246
mmu-miR-501-3p_st	229.4004	0.006309438	252.439	0.000145025
mmu-miR-721_st	85.11152	0.04064561	63.59674	0.02269715
mmu-miR-652_st	373.7676	0.000599902	776.321	1.21E-06
mmu-miR-615-3p_st	169.1551	0.01034936	279.8497	0.000926106
mmu-miR-805_st	138.0535	0.01038346	124.6132	0.01245285
mmu-miR-743a_st	118.519	0.02379829	163.0912	0.01020397
mmu-miR-181d_st	130.6767	0.009649343	392.4883	0.000377493
mmu-miR-193b_st	119.5665	0.0323408	349.024	0.000743636
mmu-miR-421_st	141.2342	0.01857322	129.8846	0.002458914
mmu-miR-466b-3p_st	228.6653	0.000259514	75.79465	0.004933881
mmu-miR-466b-3-3p_st	181.6	0.006064159	63.50697	0.01906401
mmu-miR-466c-3p_st	358.9465	0.001309796	195.3029	0.000633791
mmu-miR-466e-3p_st	306.9953	0.001785924	207.7937	0.000684763

mmu-miR-466f-3p_st	374.0187	0.000267811	409.0064	0.000210077
mmu-miR-466g_st	319.1982	0.000298034	173.231	0.00605024
mmu-miR-574-5p_st	168.899	0.009371428	282.0978	0.001338107
mmu-miR-574-3p_st	405.0378	0.00132885	338.9185	0.001162
mmu-miR-669k_st	92.06111	0.05527418	145.4735	0.01898616
mmu-miR-466i_st	269.6688	0.003132826	487.3692	3.81E-05
mmu-miR-1187_st	130.0574	0.02819728	175.2188	0.007413736
mmu-miR-467f_st	178.4973	0.009894461	197.8085	0.001718257
mmu-miR-466j_st	85.69039	0.03647212	61.61609	0.04277109
mmu-miR-1195_st	686.7464	3.29E-06	405.6268	0.000235709
mmu-miR-1196_st	1162.916	5.00E-05	216.5091	0.003531655
mmu-miR-99b-star_st	158.3648	0.01335267	277.8055	0.001239205
mmu-miR-127-star_st	262.5748	0.001810704	444.5723	0.000203406
mmu-miR-133a-star_st	131.8002	0.004082429	51.63229	0.03811923
mmu-miR-140-star_st	526.92	0.000182424	807.8068	1.38E-06
mmu-miR-150-star_st	152.771	0.006581948	15.90168	0.03193578
mmu-miR-193-star_st	77.78293	0.03497078	227.0359	0.003171266
mmu-miR-200b-star_st	134.5147	0.0188619	94.2515	0.04025601
mmu-miR-299-star_st	121.646	0.0178301	191.1412	0.004914902
mmu-miR-106b-star_st	227.666	0.002870493	305.3827	0.000705435
mmu-miR-15a-star_st	138.2123	0.02354804	174.6201	0.02182049
mmu-miR-31-star_st	96.13125	0.01492252	84.18771	0.01215605
mmu-miR-322-star_st	763.2585	9.83E-05	958.18	3.54E-07
mmu-miR-17-star_st	287.3819	0.00271912	464.9563	0.000198981
mmu-miR-28-star_st	131.6655	0.03680382	158.8175	0.008718664
mmu-miR-199b-star_st	122.4032	0.03223754	238.7198	0.00236388
mmu-miR-7a-star_st	108.6409	0.01636171	62.78576	0.05925293
mmu-miR-411-star_st	177.104	0.004445613	185.1082	0.002220236
mmu-miR-467a-star_st	340.381	0.000729368	117.8945	0.02738829
mmu-miR-485-star_st	417.9315	0.000313934	333.6454	0.000532186
mmu-miR-467b-star_st	182.3038	0.005675805	167.0405	0.006992011
mmu-miR-881-star_st	100.5125	0.02873719	96.03547	0.02589986
mmu-miR-467d-star_st	252.2046	0.001830111	134.7229	0.02112495

mmu-miR-467e-star_st	110.6878	0.03252307	124.8851	0.004765702
